# Robust Change Point Test for General Integer-Valued Time Series Models Based on Density Power Divergence

**DOI:** 10.3390/e22040493

**Published:** 2020-04-24

**Authors:** Byungsoo Kim, Sangyeol Lee

**Affiliations:** 1 Department of Statistics, Yeungnam University, Gyeongsan 38541, Korea; 2 Department of Statistics, Seoul National University, Seoul 08826, Korea; sylee@stats.snu.ac.kr

**Keywords:** integer-valued time series, one-parameter exponential family, minimum density power divergence estimator, density power divergence, robust change point test

## Abstract

In this study, we consider the problem of testing for a parameter change in general integer-valued time series models whose conditional distribution belongs to the one-parameter exponential family when the data are contaminated by outliers. In particular, we use a robust change point test based on density power divergence (DPD) as the objective function of the minimum density power divergence estimator (MDPDE). The results show that under regularity conditions, the limiting null distribution of the DPD-based test is a function of a Brownian bridge. Monte Carlo simulations are conducted to evaluate the performance of the proposed test and show that the test inherits the robust properties of the MDPDE and DPD. Lastly, we demonstrate the proposed test using a real data analysis of the return times of extreme events related to Goldman Sachs Group stock.

## 1. Introduction

Integer-valued time series models have received widespread attention from researchers and practitioners in diverse research areas. Since the works of McKenzie [1] as well as Al-Osh and Alzaid [2], integer-valued autoregressive (INAR) models have gained popularity in the analysis of correlated time series of counts. Later, as an alternative, Ferland et al. [3] proposed using Poisson integer-valued generalized autoregressive conditional heteroscedastic (INGARCH) models (see Engle [4] and Bollerslev [5]). Since then, INGARCH models have been studied by many authors, such as Fokianos et al. [6], who developed Poisson autoregressive (Poisson AR) models with nonlinear specifications for their intensity processes. The Poisson assumption on INGARCH models has been extended to include negative binomial INGARCH (NB-INGARCH) models (Davis and Wu [7] and Christou and Fokianos [8]), zero-inflated generalized Poisson INGARCH models (Zhu [9,10] and Lee et al. [11]), and one-parameter exponential distribution AR models (Davis and Liu [12]). The latter are also known as general integer-valued time series models and have been studied by, among others, Diop and Kengne [13] and Lee and Lee [14], who considered change point tests for these models.

The change point problem is a core issue in time series analysis because changes can occur in underlying model parameters owing to critical events or policy changes, and ignoring such changes can result in false conclusions. Numerous studies exist on change point analysis in time series models; refer to Kang and Lee [15] and Lee and Lee [14], and the articles cited therein, for the background and history of change points in integer-valued time series models. Lee and Lee [14] conducted a comparison study of the performance of various cumulative sum (CUSUM) tests using score vectors and residuals through the Monte Carlo simulations. In their work, the conditional maximum likelihood estimator (CMLE) is used for the parameter estimation and also the construction of the CUSUM tests. However, the CMLE is often damaged by outliers, and so is the performance of the CMLE-based CUSUM test. In general, outliers easily mislead the CUSUM test since they can be mistakenly taken for abrupt changes; in the opposite, they can misidentify change points in their presence on time series. Among the robust estimation methods, we adopt the minimum density power divergence estimator (MDPDE) approach—proposed by Basu et al. [16]—as a remedy and propose to use the density power divergence (DPD)-based test as a robust change point test.

The MDPDE method is well known for consistently making robust inferences in various situations, and the trade-off between efficiency and robustness is managed via the tuning parameter. Basu et al. [16] introduced the MDPDE using the independent and identically distributed observations, and later, Ghosh and Basu [17] extended their method to the independent but not identically distributed samples. For earlier works in the context of time series, see Lee and Song [18], Kim and Lee [19], Kang and Lee [20], and Kim and Lee [21], who deal with the MDPDE for GARCH models, multivariate times series, and (zero-inflated) Poisson AR models. Kim and Lee [22] demonstrated that the MDPDE for general integer-valued time series models has strong robust properties, with little loss in asymptotic efficiency relative to the CMLE. This motivates us to use the MDPDE to construct a robust change point test for general integer-valued time series models. More precisely, we anticipate that the robust property of the MDPDE would be inherited to the proposed change point test, so that the influence of outliers should be reduced when performing a parameter change test in the presence of outliers. Although the problem of testing for a parameter change in integer-valued time series models has been investigated by many researchers, the testing procedure for observations with outliers has not been widely studied. This motivates us to develop a MDPDE-based robust change point test for general integer-valued time series models.

Kang and Song [23] proposed an estimate-based robust CUSUM test that uses the MDPDE to detect parameter changes in Poisson AR models. However, this type of test is known to suffer from severe size distortions, especially when the true parameter lies at the boundary of the parameter space. Thus, we use the test deduced based on an empirical version of the DPD, which is the objective function of the MDPDE. Song and Kang [24] and Kang and Song [25] applied DPD-based change point tests in GARCH models and Poisson AR models, respectively. However, the DPD approach basically shares the same spirit as the score-based CUSUM test of Lee and Lee [14] (see Remark 3 in Section 2.2), in that both are based on derivatives of objective functions. Thus, the idea is easily adapted to one-parameter exponential family AR models. As for a parameter change test for independent samples based on divergence measures, see Batsidis et al. [26,27], who consider the ϕ-divergence as a measure. We also refer to Martín and Pardo [28], who point out the importance of a Wald-type test based on DPD in dealing with the change point problem.

Monte Carlo simulations are conducted to evaluate the performance of the proposed test. Here, we compare the DPD-based test and the score-based CUSUM test to demonstrate the superiority of the proposed test in the presence of outliers. Then, we provide a real data analysis of the return times of extreme events related to Goldman Sachs Group (GS) stock to illustrate the proposed test. The paper proceeds as follows. Section 2 constructs the DPD-based change point test for general integer-valued time series models, and states its weak convergence theorem. Section 3 presents a simulation study and a real data analysis. Section 4 concludes the paper. All proofs are provided in the Appendix A.

## 2. Construction of the MDPDE and Change Point Test

### 2.1. MDPDE for General Integer-Valued Time Series Models

Let $Y_{1},Y_{2},\ldots$ be the observations generated from general integer-valued time series models with the conditional distribution of the one-parameter exponential family:

(1) $$\begin{array}{c} Y_{t}|F_{t-1}∼p\left(y|\eta_{t}\right),\;\;\;X_{t}:=E\left(Y_{t}|F_{t-1}\right)=f_{\theta }\left(X_{t-1},Y_{t-1}\right), \end{array}$$

where $F_{t-1}$ is a σ-field generated by $Y_{t-1},Y_{t-2},\ldots$ and $f_{\theta }\left(x,y\right)$ is a non-negative bivariate function defined on $\left[0,\infty \right)\times N_{0},\;N_{0}=N\cup \left\{0\right\}$, depending on the parameter $\theta \in \Theta \subset R^{d}$, and satisfies $\inf_{\theta \in \Theta }f_{\theta }\left(x,y\right)\geq x^{*}$ for some $x^{*}>0$ for all x,y. Here, p(·|·) is a probability mass function, given by

$$\begin{array}{c} p(y|\eta )=\exp\{\eta y-A(\eta )\}h(y),\;\;\;y\geq 0, \end{array}$$

where η is the natural parameter and A(η) and h(y) are known functions. This distribution family includes several famous discrete distributions, such as the Poisson, negative binomial, and binomial distributions. If $B\left(\eta \right)=A^{\prime }\left(\eta \right)$, $B(\eta_{t})$ and $B^{\prime }\left(\eta_{t}\right)$ become the conditional mean and variance of $Y_{t}$, and $X_{t}=B\left(\eta_{t}\right)$. The derivative of A(η) exists for the exponential family; see Lehmann and Casella [29]. Since $B^{\prime }\left(\eta_{t}\right)=Var\left(Y_{t}|F_{t-1}\right)>0$, B(η) is strictly increasing, and since $B\left(\eta_{t}\right)=E\left(Y_{t}|F_{t-1}\right)>0$, A(η) is also strictly increasing. To emphasize the role of θ, we also use $X_{t}\left(\theta \right)$ and $\eta_{t}\left(\theta \right)=B^{-1}\left(X_{t}\left(\theta \right)\right)$ to stand for $X_{t}$ and $\eta_{t}$, respectively.

Davis and Liu [12] showed that the assumption below ensures the strict stationarity and ergodicity of $\left\{\left(X_{t},Y_{t}\right)\right\}$:

**(A0)**  For all $x,x^{\prime }\geq 0$ and $y,y^{\prime }\in N_{0}$,
  $$\begin{array}{c} \underset{\theta \in \Theta }{\sup}|f_{\theta }\left(x,y\right)-f_{\theta }\left(x^{\prime },y^{\prime }\right)|\leq \omega_{1}|x-x^{\prime }|+\omega_{2}\left|y-y^{\prime }\right|, \end{array}$$
  where $\omega_{1},\omega_{2}\geq 0$ satisfy $\omega_{1}+\omega_{2}<1$.

They also demonstrated that there exists a measurable function $f_{\infty }^{\theta }:\;N_{0}^{\infty }\to \left[0,\infty \right)$, such that $X_{t}\left(\theta \right)=f_{\infty }^{\theta }\left(Y_{t-1},Y_{t-2},\ldots \right)$ almost surely (a.s.).

Meanwhile, the DPD $d_{\alpha }$ between two density functions *g* and *h* is defined as

$$d_{\alpha }\left(g,h\right):=\left\{\begin{array}{cc} \int \{g^{1+\alpha }\left(y\right)-\left(1+\frac{1}{\alpha }\right)h\left(y\right)g^{\alpha }\left(y\right)+\frac{1}{\alpha }h^{1+\alpha }\left(y\right)\}dy, & \alpha >0,\\ \int h(y)(\log h(y)-\log g(y))dy, & \alpha =0. \end{array}\right.$$

For a parametric family $\left\{G_{\theta },\;\theta \in \Theta \right\}$ with densities given by $\left\{g_{\theta }\right\}$ and a distribution *H* with density *h*, the minimum DPD functional $T_{\alpha }\left(H\right)$ is defined by $d_{\alpha }\left(h,g_{T_{\alpha }\left(H\right)}\right)=\min_{\theta \in \Theta }d_{\alpha }\left(h,g_{\theta }\right).$ In particular, if $H=G_{\theta_{0}}\in \left\{G_{\theta }\right\}$, $T_{\alpha }\left(G_{\theta_{0}}\right)=\theta_{0}$. Then, given a random sample $Y_{1},\ldots ,Y_{n}$ with unknown density *h*, the MDPDE is defined by

$${\hat{\theta }}_{\alpha ,n}=\underset{\theta \in \Theta }{\mathrm{argmin}}L_{\alpha ,n}\left(\theta \right),$$

where $L_{\alpha ,n}\left(\theta \right)=\frac{1}{n}\sum_{t=1}^{n}l_{\alpha ,t}\left(\theta \right)$ and

$$l_{\alpha ,t}\left(\theta \right)=\left\{\begin{array}{cc} \int g_{\theta }^{1+\alpha }\left(y\right)dy-\left(1+\frac{1}{\alpha }\right)g_{\theta }^{\alpha }\left(Y_{t}\right), & \alpha >0,\\ -\log g_{\theta }\left(Y_{t}\right), & \alpha =0. \end{array}\right.$$

When α=0 and 1, the MDPDE becomes the MLE and the $L^{2}$-distance estimator, respectively. Basu et al. [16] revealed that ${\hat{\theta }}_{\alpha ,n}$ is consistent for $T_{\alpha }\left(H\right)$ and asymptotically normal. Furthermore, the estimator is robust against outliers, but still exhibits high efficiency when the true distribution belongs to a parametric family $\left\{G_{\theta }\right\}$ and α is close to zero. The tuning parameter α controls the trade-off between robustness and asymptotic efficiency. A large α escalates the robustness while a small α yields greater efficiency. The conditional version of the MDPDE is defined similarly (cf. Section 2 of Kim and Lee [22]).

For $Y_{1},\ldots ,Y_{n}$ generated from (1), the MDPDE for general integer-valued time series models is defined as

(2) $$\begin{array}{c} {\hat{\theta }}_{\alpha ,n}=\underset{\theta \in \Theta }{\mathrm{argmin}}{\tilde{L}}_{\alpha ,n}\left(\theta \right)=\underset{\theta \in \Theta }{\mathrm{argmin}}\frac{1}{n}\sum_{t=1}^{n}{\tilde{l}}_{\alpha ,t}\left(\theta \right), \end{array}$$

where

(3) $$\begin{array}{cc} {\tilde{l}}_{\alpha ,t}\left(\theta \right) & =\left\{\begin{array}{cc} \sum_{y=0}^{\infty }p^{1+\alpha }\left(y|{\tilde{\eta }}_{t}\left(\theta \right)\right)-\left(1+\frac{1}{\alpha }\right)p^{\alpha }\left(Y_{t}|{\tilde{\eta }}_{t}\left(\theta \right)\right), & \alpha >0,\\ -\log p(Y_{t}|{\tilde{\eta }}_{t}\left(\theta \right)), & \alpha =0, \end{array}\right. \end{array}$$

and ${\tilde{\eta }}_{t}\left(\theta \right)=B^{-1}\left({\tilde{X}}_{t}\left(\theta \right)\right)$ is updated recursively using the following equations:

$$\begin{array}{c} {\tilde{X}}_{t}\left(\theta \right)=f_{\theta }\left({\tilde{X}}_{t-1}\left(\theta \right),Y_{t-1}\right),\;t=2,3,\ldots ,\;\;{\tilde{X}}_{1}\left(\theta \right)={\tilde{X}}_{1}, \end{array}$$

with an arbitrarily chosen initial value ${\tilde{X}}_{1}$. The MDPDE with α=0 becomes the CMLE from (3).

Kim and Lee [22] showed that under the regularity conditions **(A0)**–**(A9)** stated below, the MDPDE is strongly consistent and asymptotically normal. Conditions **(A10)** and **(A11)** are imposed to derive the limiting null distribution of the DPD-based change point test in Section 2.2. Below, *V* and ρ∈(0,1) represent a generic integrable random variable and a constant, respectively; the symbol ∥·∥ denotes the $L^{2}$-norm for matrices and vectors; and E(·) is taken under $\theta_{0}$, where $\theta_{0}$ denotes the true value of θ.

**(A1)**  $\theta_{0}$ is an interior point in the compact parameter space $\Theta \subset R^{d}$.
**(A2)**  $E{\left(\sup_{\theta \in \Theta }X_{1}\left(\theta \right)\right)}^{4}<\infty$.
**(A3)**  $\inf_{\theta \in \Theta }\inf_{0\leq \delta \leq 1}B^{\prime }\left(\left(1-\delta \right)\eta_{t}\left(\theta \right)+\delta {\tilde{\eta }}_{t}\left(\theta \right)\right)\geq \underline{c}$ for some $\underline{c}>0$.
**(A4)**  $EY_{1}^{4}<\infty$.
**(A5)**  If there exists t≥1, such that $X_{t}\left(\theta \right)=X_{t}\left(\theta_{0}\right)$ a.s., then $\theta =\theta_{0}$.
**(A6)**  $\sup_{\theta \in \Theta }\sup_{0\leq \delta \leq 1}\left|\frac{B^{\prime \prime }\left(\left(1-\delta \right)\eta_{t}\left(\theta \right)+\delta {\tilde{\eta }}_{t}\left(\theta \right)\right)}{B^{\prime }{\left(\left(1-\delta \right)\eta_{t}\left(\theta \right)+\delta {\tilde{\eta }}_{t}\left(\theta \right)\right)}^{5/2}}\right|\leq K$ for some K>0.
**(A7)**  The mapping $\theta \mapsto f_{\infty }^{\theta }$ is twice continuously differentiable with respect to θ, and satisfies
  $$\begin{array}{c} E{\left(\underset{\theta \in \Theta }{\sup}∥\frac{\partial f_{\infty }^{\theta }\left(Y_{0},Y_{-1},\ldots \right)}{\partial \theta }∥\right)}^{4}<\infty \;\;\;\;\;\mathrm{and}\;\;\;E{\left(\underset{\theta \in \Theta }{\sup}∥\frac{\partial^{2}f_{\infty }^{\theta }\left(Y_{0},Y_{-1},\ldots \right)}{\partial \theta \partial \theta^{T}}∥\right)}^{2}<\infty . \end{array}$$
**(A8)**  $\sup_{\theta \in \Theta }∥\frac{\partial {\tilde{X}}_{t}\left(\theta \right)}{\partial \theta }-\frac{\partial X_{t}\left(\theta \right)}{\partial \theta }∥\leq V\rho^{t}$ a.s.
**(A9)**  $\nu^{T}\frac{\partial X_{t}\left(\theta_{0}\right)}{\partial \theta }=0$ a.s. implies ν=0.
**(A10)**  $\sup_{\theta \in \Theta }∥\frac{\partial^{2}{\tilde{X}}_{t}\left(\theta \right)}{\partial \theta \partial \theta^{T}}-\frac{\partial^{2}X_{t}\left(\theta \right)}{\partial \theta \partial \theta^{T}}∥\leq V\rho^{t}$ a.s.
**(A11)**  $\sup_{\theta \in \Theta }\sup_{0\leq \delta \leq 1}\left|\frac{B^{\left(3\right)}\left(\left(1-\delta \right)\eta_{t}\left(\theta \right)+\delta {\tilde{\eta }}_{t}\left(\theta \right)\right)}{B^{\prime }{\left(\left(1-\delta \right)\eta_{t}\left(\theta \right)+\delta {\tilde{\eta }}_{t}\left(\theta \right)\right)}^{4}}\right|\leq M$ for some M>0.

**Proposition** **1.** *Under* **(A0)** *–* **(A5)** *, ${\hat{\theta }}_{\alpha ,n}⟶\theta_{0}$ a.s. as n→∞, and further, under* **(A0)** *–* **(A9)** *,*

$$\sqrt{n}\left({\hat{\theta }}_{\alpha ,n}-\theta_{0}\right)\overset{d}{⟶}N\left(0,J_{\alpha }^{-1}K_{\alpha }J_{\alpha }^{-1}\right)\;\;as\;\;n\to \infty ,$$

*where*

$$J_{\alpha }=-E\left(\frac{\partial^{2}l_{\alpha ,t}\left(\theta_{0}\right)}{\partial \theta \partial \theta^{T}}\right),\;K_{\alpha }=E\left(\frac{\partial l_{\alpha ,t}\left(\theta_{0}\right)}{\partial \theta }\frac{\partial l_{\alpha ,t}\left(\theta_{0}\right)}{\partial \theta^{T}}\right)$$

*and $l_{\alpha ,t}\left(\theta \right)$ is defined by substituting $\eta_{t}\left(\theta \right)$ for ${\tilde{\eta }}_{t}\left(\theta \right)$ in (3).*

**Remark** **1.** *In our empirical study, discussed in Section 3.2, we select an optimal α using the method of Warwick [30] and Warwick and Jones [31]. We choose α that minimizes the trace of the estimated asymptotic mean squared error ($\hat{AMSE}$):*

$$\hat{AMSE}=\left({\hat{\theta }}_{\alpha ,n}-{\hat{\theta }}_{1,n}\right){\left({\hat{\theta }}_{\alpha ,n}-{\hat{\theta }}_{1,n}\right)}^{T}+\hat{As.var}\left({\hat{\theta }}_{\alpha ,n}\right),$$

*where ${\hat{\theta }}_{1,n}$ is the MDPDE with α=1 and $\hat{As.var}\left({\hat{\theta }}_{\alpha ,n}\right)$ is the estimate of the asymptotic variance of ${\hat{\theta }}_{\alpha ,n}$, computed as*

$$\hat{As.var}\left({\hat{\theta }}_{\alpha ,n}\right)={\left(\sum_{t=1}^{n}\frac{\partial^{2}{\tilde{l}}_{\alpha ,t}\left({\hat{\theta }}_{\alpha ,n}\right)}{\partial \theta \partial \theta^{T}}\right)}^{-1}\left(\sum_{t=1}^{n}\frac{\partial {\tilde{l}}_{\alpha ,t}\left({\hat{\theta }}_{\alpha ,n}\right)}{\partial \theta }\frac{\partial {\tilde{l}}_{\alpha ,t}\left({\hat{\theta }}_{\alpha ,n}\right)}{\partial \theta^{T}}\right){\left(\sum_{t=1}^{n}\frac{\partial^{2}{\tilde{l}}_{\alpha ,t}\left({\hat{\theta }}_{\alpha ,n}\right)}{\partial \theta \partial \theta^{T}}\right)}^{-1}.$$

**Remark** **2.** *Instead of* **(A6)** *, Kim and Lee [22] assumed*

$$\underset{\theta \in \Theta }{\sup}\underset{0\leq \delta \leq 1}{\sup}\left|\frac{B^{\prime \prime }\left(\left(1-\delta \right)\eta_{t}\left(\theta \right)+\delta {\tilde{\eta }}_{t}\left(\theta \right)\right)}{B^{\prime }{\left(\left(1-\delta \right)\eta_{t}\left(\theta \right)+\delta {\tilde{\eta }}_{t}\left(\theta \right)\right)}^{3}}\right|\leq K\;forsome\;K>0$$

*to prove Proposition 1. Note that this condition is satisfied directly if* **(A3)** *and* **(A6)** *hold. In our study, we alter the above condition to* **(A6)** *to prove Lemma A1 in the Appendix A, which is needed to obtain the limiting null distribution of the DPD-based change point test in Section 2.2.*

The following INGARCH(1,1) models are typical examples of general integer-valued time series models:

$$\begin{array}{c} Y_{t}|F_{t-1}∼p\left(y|\eta_{t}\right),\;\;\;X_{t}=d+aX_{t-1}+bY_{t-1}, \end{array}$$

where $X_{t}=B\left(\eta_{t}\right)=E\left(Y_{t}|F_{t-1}\right)$, $\theta ={\left(d,a,b\right)}^{T}\in \Theta \subset \left(0,\infty \right)\times {\left[0,\infty \right)}^{2}$ with a+b<1, and Θ is compact. Condition **(A0)** trivially holds, and the process $\left\{\left(X_{t},Y_{t}\right),\;t\geq 1\right\}$ has a strictly stationary and ergodic solution. Condition **(A1)** can be replaced with the following:

**(A1)′**  The true parameter $\theta_{0}$ lies in a compact neighborhood $\Theta \in R_{+}^{3}$ of $\theta_{0}$, where
  $$\begin{array}{c} \Theta \in \left\{\theta ={\left(d,a,b\right)}^{T}\in R_{+}^{3}:0<d_{L}\leq d\leq d_{U},\;\epsilon \leq a+b\leq 1-\epsilon \right\}\;\mathrm{for}\;\mathrm{some}\;d_{L},d_{U},\epsilon >0. \end{array}$$

Moreover, we can express

$$\begin{array}{c} X_{t}\left(\theta \right)=\frac{d}{1-a}+b\sum_{k=0}^{\infty }a^{k}Y_{t-k-1}\;\;\;\mathrm{and}\;\;\;{\tilde{X}}_{t}\left(\theta \right)=\frac{d}{1-a}+b\sum_{k=0}^{t-2}a^{k}Y_{t-k-1}, \end{array}$$

where the initial value ${\tilde{X}}_{1}$ is taken as d/(1−a) for simplicity. Based on the above and **(A4)**, the conditions **(A2)**, **(A5)**, and **(A7)–(A10)** are all satisfied for INGARCH(1,1) models, as proven by Theorem 3 of Kang and Lee [15]. Kim and Lee [22] showed recently that the following Poisson and negative binomial INGARCH(1,1) models satisfy **(A3)** and **(A4)**. Furthermore, following the arguments presented in Section 3.2 of their study, **(A6)** holds for these models as well. Below, we show that **(A11)** holds for Poisson and negative binomial INGARCH(1,1) models.

• *Poisson INGARCH(1,1) model:*

$$\begin{array}{c} Y_{t}|F_{t-1}∼\mathrm{Poisson}\left(X_{t}\right),\;\;\;X_{t}=d+aX_{t-1}+bY_{t-1}. \end{array}$$

In this model, $\eta_{t}\left(\theta \right)=\log\left(X_{t}\left(\theta \right)\right)$ and $A\left(\eta_{t}\left(\theta \right)\right)=e^{\eta_{t}\left(\theta \right)}$. Since $B^{\prime }\left(\eta \right)=B^{\left(3\right)}\left(\eta \right)$, **(A11)** holds owing to **(A3)**.

• *NB-INGARCH(1,1) model:*

$$\begin{array}{c} Y_{t}|F_{t-1}∼NB\left(r,p_{t}\right),\;\;\;X_{t}=\frac{r(1-p_{t})}{p_{t}}=d+aX_{t-1}+bY_{t-1}, \end{array}$$

where NB(r,p) denotes a negative binomial distribution with parameters r∈N and p∈(0,1). To be more specific, it counts the number of failures before the *r*-th success occurs in a sequence of Bernoulli trials with success probability *p*. Here, *r* is assumed to be known. In this model, $\eta_{t}\left(\theta \right)=\log\left(X_{t}\left(\theta \right)/\left(X_{t}\left(\theta \right)+r\right)\right)$ and $A\left(\eta_{t}\left(\theta \right)\right)=r\log\left(r/\left(1-e^{\eta_{t}\left(\theta \right)}\right)\right)$. From the fact that $B^{\prime }\left(\eta \right)=re^{\eta }/{\left(1-e^{\eta }\right)}^{2}$ and $B^{\left(3\right)}\left(\eta \right)=re^{\eta }\left(e^{2\eta }+4e^{\eta }+1\right)/{\left(1-e^{\eta }\right)}^{4}$, we have $B^{\left(3\right)}\left(\eta \right)/B^{\prime }{\left(\eta \right)}^{4}={\left(1-e^{\eta }\right)}^{4}\left(e^{2\eta }+4e^{\eta }+1\right)/r^{3}e^{3\eta }$, which is positive and strictly decreasing on η<0. Moreover, since $d_{L}/\left(d_{L}+r\right)\leq e^{\eta_{t}\left(\theta \right)}<1$, it holds that

$$\begin{array}{c} \frac{B^{\left(3\right)}\left(\eta_{t}\left(\theta \right)\right)}{B^{\prime }{\left(\eta_{t}\left(\theta \right)\right)}^{4}}\leq \frac{6{\left(1-d_{L}/\left(d_{L}+r\right)\right)}^{4}}{r^{3}{\left(d_{L}/\left(d_{L}+r\right)\right)}^{3}}=\frac{6r}{d_{L}^{3}\left(d_{L}+r\right)} \end{array}$$

and $B^{\left(3\right)}\left({\tilde{\eta }}_{t}\left(\theta \right)\right)/B^{\prime }{\left({\tilde{\eta }}_{t}\left(\theta \right)\right)}^{4}$ also has the same upper bound. Hence, **(A11)** is satisfied.

In addition to the above models, general integer-valued time series models also include nonlinear models, such as the integer-valued threshold GARCH (INTGARCH) model:

$$\begin{array}{c} Y_{t}|F_{t-1}∼Poisson\left(X_{t}\right),\;\;\;X_{t}=d+aX_{t-1}+b_{1}\max\left(Y_{t-1}-l,0\right)+b_{2}\min\left(Y_{t-1},l\right), \end{array}$$

where $\theta ={\left(d,a,b_{1},b_{2}\right)}^{T}\in \Theta \subset \left(0,\infty \right)\times {\left[0,\infty \right)}^{3}$ with $a+\max(b_{1},b_{2})<1$, Θ is compact, and *l* is a non-negative integer value. For more details, see Remark 3 in Kim and Lee [22].

### 2.2. DPD-Based Change Point Test

As a robust test for parameter changes in general integer-valued time series models, we propose a DPD-based test for the following hypotheses:

$$H_{0}:\theta \;\;\;\mathrm{does}\;\mathrm{not}\;\mathrm{change}\;\mathrm{over}\;\;\;Y_{1},\cdots ,Y_{n}\;\;\;\mathrm{vs}.\;\;\;H_{1}:\;\mathrm{not}\;\;H_{0}.$$

To construct the test, we employ the objective function of the MDPDE. That is, our test is constructed using the empirical version of the DPD. Let ${\tilde{L}}_{\alpha ,n}$ be that in (2). To implement our test, we employ the following test statistic:

$${\hat{T}}_{n}^{\alpha }:=\max_{1\leq k\leq n}\frac{k^{2}}{n}\frac{\partial {\tilde{L}}_{\alpha ,k}\left({\hat{\theta }}_{\alpha ,n}\right)}{\partial \theta^{T}}{\hat{K}}_{\alpha }^{-1}\frac{\partial {\tilde{L}}_{\alpha ,k}\left({\hat{\theta }}_{\alpha ,n}\right)}{\partial \theta },$$

where

$${\hat{K}}_{\alpha }=\frac{1}{n}\sum_{t=1}^{n}\frac{\partial {\tilde{l}}_{\alpha ,t}\left({\hat{\theta }}_{\alpha ,n}\right)}{\partial \theta }\frac{\partial {\tilde{l}}_{\alpha ,t}\left({\hat{\theta }}_{\alpha ,n}\right)}{\partial \theta^{T}}$$

is a consistent estimator of $K_{\alpha }$. For the consistency of ${\hat{K}}_{\alpha }$, see Lemma A5 in Appendix A.

Using the mean value theorem (MVT), we have the following, for each s∈[0,1],

(4) $$\begin{array}{c} \frac{\left[ns\right]}{\sqrt{n}}\frac{\partial {\tilde{L}}_{\alpha ,[ns]}\left({\hat{\theta }}_{\alpha ,n}\right)}{\partial \theta }=\frac{\left[ns\right]}{\sqrt{n}}\frac{\partial {\tilde{L}}_{\alpha ,[ns]}\left(\theta_{0}\right)}{\partial \theta }+\frac{\left[ns\right]}{n}\frac{\partial^{2}{\tilde{L}}_{\alpha ,[ns]}\left(\theta_{\alpha ,n,s}^{*}\right)}{\partial \theta \partial \theta^{T}}\sqrt{n}\left({\hat{\theta }}_{\alpha ,n}-\theta_{0}\right), \end{array}$$

where $\theta_{\alpha ,n,s}^{*}$ is an intermediate point between ${\hat{\theta }}_{\alpha ,n}$ and $\theta_{0}$. From $\partial {\tilde{L}}_{\alpha ,n}\left({\hat{\theta }}_{\alpha ,n}\right)/\partial \theta =0$, we obtain that, for s=1,

$$\begin{array}{c} 0=\sqrt{n}\frac{\partial {\tilde{L}}_{\alpha ,n}\left(\theta_{0}\right)}{\partial \theta }+\frac{\partial^{2}{\tilde{L}}_{\alpha ,n}\left(\theta_{\alpha ,n,1}^{*}\right)}{\partial \theta \partial \theta^{T}}\sqrt{n}\left({\hat{\theta }}_{\alpha ,n}-\theta_{0}\right). \end{array}$$

Furthermore, since $J_{\alpha }$ is nonsingular (cf. proof of Lemma 7 in Kim and Lee [22]), this can be expressed as

$$\begin{array}{cc} \sqrt{n}\left({\hat{\theta }}_{\alpha ,n}-\theta_{0}\right) & =J_{\alpha }^{-1}\sqrt{n}\frac{\partial {\tilde{L}}_{\alpha ,n}\left(\theta_{0}\right)}{\partial \theta }+J_{\alpha }^{-1}\frac{\partial^{2}{\tilde{L}}_{\alpha ,n}\left(\theta_{\alpha ,n,1}^{*}\right)}{\partial \theta \partial \theta^{T}}\sqrt{n}\left({\hat{\theta }}_{\alpha ,n}-\theta_{0}\right)+\sqrt{n}\left({\hat{\theta }}_{\alpha ,n}-\theta_{0}\right)\\ & =J_{\alpha }^{-1}\sqrt{n}\frac{\partial {\tilde{L}}_{\alpha ,n}\left(\theta_{0}\right)}{\partial \theta }+J_{\alpha }^{-1}\left(\frac{\partial^{2}{\tilde{L}}_{\alpha ,n}\left(\theta_{\alpha ,n,1}^{*}\right)}{\partial \theta \partial \theta^{T}}+J_{\alpha }\right)\sqrt{n}\left({\hat{\theta }}_{\alpha ,n}-\theta_{0}\right). \end{array}$$

Substituting the above into (4) yields

(5) $$\begin{array}{cc} \frac{\left[ns\right]}{\sqrt{n}}\frac{\partial {\tilde{L}}_{\alpha ,[ns]}\left({\hat{\theta }}_{\alpha ,n}\right)}{\partial \theta }= & \frac{\left[ns\right]}{\sqrt{n}}\frac{\partial {\tilde{L}}_{\alpha ,[ns]}\left(\theta_{0}\right)}{\partial \theta }+\frac{\left[ns\right]}{n}\frac{\partial^{2}{\tilde{L}}_{\alpha ,[ns]}\left(\theta_{\alpha ,n,s}^{*}\right)}{\partial \theta \partial \theta^{T}}J_{\alpha }^{-1}\sqrt{n}\frac{\partial {\tilde{L}}_{\alpha ,n}\left(\theta_{0}\right)}{\partial \theta }\\ & +\frac{\left[ns\right]}{n}\frac{\partial^{2}{\tilde{L}}_{\alpha ,[ns]}\left(\theta_{\alpha ,n,s}^{*}\right)}{\partial \theta \partial \theta^{T}}J_{\alpha }^{-1}\left(\frac{\partial^{2}{\tilde{L}}_{\alpha ,n}\left(\theta_{\alpha ,n,1}^{*}\right)}{\partial \theta \partial \theta^{T}}+J_{\alpha }\right)\sqrt{n}\left({\hat{\theta }}_{\alpha ,n}-\theta_{0}\right). \end{array}$$

In Appendix A, we show that the first two terms on the right-hand side of (5) converge weakly to $K_{\alpha }^{1/2}B_{d}^{o}\left(s\right)$, where $B_{d}^{o}$ is a *d*-dimensional standard Brownian bridge and the last term is asymptotically negligible. Therefore, we obtain the following theorem.

**Theorem** **1.** *Suppose that conditions* **(A0)** *–* **(A11)** *hold. Then, under $H_{0}$, we have*

$$K_{\alpha }^{-1/2}\frac{\left[ns\right]}{\sqrt{n}}\frac{\partial {\tilde{L}}_{\alpha ,[ns]}\left({\hat{\theta }}_{\alpha ,n}\right)}{\partial \theta }\overset{w}{⟶}B_{d}^{o}\left(s\right).$$

*Therefore,*

$${\hat{T}}_{n}^{\alpha }\overset{d}{⟶}\underset{0\leq s\leq 1}{\sup}{∥B_{d}^{o}\left(s\right)∥}^{2}.$$

We reject $H_{0}$ if ${\hat{T}}_{n}^{\alpha }$ is large; see Table 1 of Lee et al. [32] for the critical values. When a change point is detected, its location is estimated as

$$\underset{1\leq k\leq n}{\mathrm{argmax}}\frac{k^{2}}{n}\frac{\partial {\tilde{L}}_{\alpha ,k}\left({\hat{\theta }}_{\alpha ,n}\right)}{\partial \theta^{T}}{\hat{K}}_{\alpha }^{-1}\frac{\partial {\tilde{L}}_{\alpha ,k}\left({\hat{\theta }}_{\alpha ,n}\right)}{\partial \theta }.$$

**Remark** **3.** *The proposed test ${\hat{T}}_{n}^{\alpha }$ with α=0 is the same as the score-vector-based CUSUM test proposed by Lee and Lee [14], given by*

$$\begin{array}{c} {\hat{T}}_{n}^{score}=\max_{1\leq k\leq n}\frac{1}{n}\left(\sum_{t=1}^{k}\frac{\partial {\tilde{l}}_{0,t}\left({\hat{\theta }}_{0,n}\right)}{\partial \theta^{T}}\right){\hat{I}}_{n}^{-1}\left(\sum_{t=1}^{k}\frac{\partial {\tilde{l}}_{0,t}\left({\hat{\theta }}_{0,n}\right)}{\partial \theta }\right), \end{array}$$

*where ${\tilde{l}}_{0,t}\left(\theta \right)$ is defined in (3), ${\hat{\theta }}_{0,n}$ is the CMLE, and ${\hat{I}}_{n}=n^{-1}\sum_{t=1}^{n}\partial^{2}{\tilde{l}}_{0,t}\left({\hat{\theta }}_{0,n}\right)/\partial \theta \partial \theta^{T}$. In the next section, we compare the performance of ${\hat{T}}_{n}^{\alpha }$ with that of ${\hat{T}}_{n}^{score}$ in the presence of outliers.*

## 3. Empirical Studies

### 3.1. Simulation

In this section, we evaluate the performance of the proposed test ${\hat{T}}_{n}^{\alpha }$ (with α>0) through simulations, focusing on the comparison with ${\hat{T}}_{n}^{score}$. First, we consider the Poisson INGARCH models:

(6) $$\begin{array}{c} Y_{t}|F_{t-1}∼\mathrm{Poisson}\left(X_{t}\right),\;\;\;X_{t}=d+aX_{t-1}+bY_{t-1}, \end{array}$$

where $X_{1}$ is set to 0 for the data generation and ${\tilde{X}}_{1}$ is set as the sample mean of the data. The sample sizes considered are n=500 and 1000, with 1000 repetitions for each simulation. For the comparison, we examine the empirical size and power at the nominal level of 0.05, which has a corresponding critical value of 3.004. To calculate the empirical size and power for each test, we consider cases with θ=(d,a,b)=(1,0.2,0.2),(1,0.2,0.4),(1,0.2,0.7) and those in which θ=(d,a,b)=(1,0.2,0.2) changes to $\theta^{\prime }=\left(d^{\prime },a^{\prime },b^{\prime }\right)=\left(1.5,0.2,0.2\right),\;\left(1,0.4,0.2\right),\;\left(1,0.2,0.4\right)$ at the middle time t=[n/2], respectively.

Table 1 presents the results when the data are not contaminated by outliers, showing that both tests (${\hat{T}}_{n}^{score}$ and ${\hat{T}}_{n}^{\alpha }$) exhibit reasonable size, even when a+b is close to 1. When n=500, ${\hat{T}}_{n}^{score}$ outperforms ${\hat{T}}_{n}^{\alpha }$ in terms of power; however, as the sample size increases to n=1000, ${\hat{T}}_{n}^{\alpha }$ exhibits similar power to that of ${\hat{T}}_{n}^{score}$, particularly when α is small. The power of ${\hat{T}}_{n}^{\alpha }$ tends to decrease as α increases, confirming that an MDPDE with large α results in a loss of efficiency.

To evaluate the robustness of the proposed test, we assume that contaminated data $Y_{c,t}$ are observed instead of $Y_{t}$ in (6) (cf. Fried et al. [33]):

(7) $$\begin{array}{c} Y_{c,t}=Y_{t}+P_{t}Y_{o,t}, \end{array}$$

where $P_{t}$ are independent and identically distributed (iid) Bernoulli random variables with success probability *p* and $Y_{o,t}$ are iid Poisson random variables with mean γ. We assume that $Y_{t}$, $P_{t}$, and $Y_{o,t}$ are all independent. In this simulation, we consider the cases p=0.01,0.03 and γ=5,10. The results are reported in Table 2, Table 3, Table 4 and Table 5, showing that ${\hat{T}}_{n}^{score}$ suffers from size distortions that become more severe as either *p* or γ increase. In contrast, ${\hat{T}}_{n}^{\alpha }$ compensates for this defect remarkably well, yielding comparable power to that of ${\hat{T}}_{n}^{score}$ when n=1000. This indicates that as more data are contaminated by outliers, ${\hat{T}}_{n}^{\alpha }$ increasingly outperforms ${\hat{T}}_{n}^{score}$.

Next, we consider the following NB-INGARCH(1,1) models:

(8) $$\begin{array}{c} Y_{t}|F_{t-1}∼NB\left(r,p_{t}\right),\;\;\;X_{t}=\frac{r(1-p_{t})}{p_{t}}=d+aX_{t-1}+bY_{t-1}, \end{array}$$

where $X_{1}$ and ${\tilde{X}}_{1}$ are 0 and the sample mean of the data, respectively. We set r=10, and use the same parameter settings as in the Poisson INGARCH model case. In order to evaluate the robustness of the test, we observe contaminated data $Y_{c,t}$, as in (7), where $Y_{t}$ are generated from (8), $P_{t}$ are iid Bernoulli random variables with success probability *p*, and $Y_{o,t}$ are iid NB(10,κ) random variables. We consider the cases p=0.01,0.03 and κ=0.6,0.5. The results are reported in Table 6, Table 7, Table 8, Table 9 and Table 10, showing similar results to those in Table 1, Table 2, Table 3, Table 4 and Table 5. Our findings show that the DPD-based test performs reasonably well in terms of both size and power, regardless of the existence of outliers. In addition, we confirm that the proposed test outperforms the score-based CUSUM test when the data are contaminated by outliers.

### 3.2. Real Data Analysis

In this section, we demonstrate the validity of ${\hat{T}}_{n}^{\alpha }$ using a real data analysis. To this end, we analyze the return times of extreme events related to GS stock, which are constructed based on the daily log-returns for the period of 5 May 1999 to 15 March 2012. Davis and Liu [12] and Kim and Lee [22] previously investigated this data set in their works on geometric INGARCH(1,1) models (i.e., NB-INGARCH(1,1) models with r=1).

**Figure 1:**
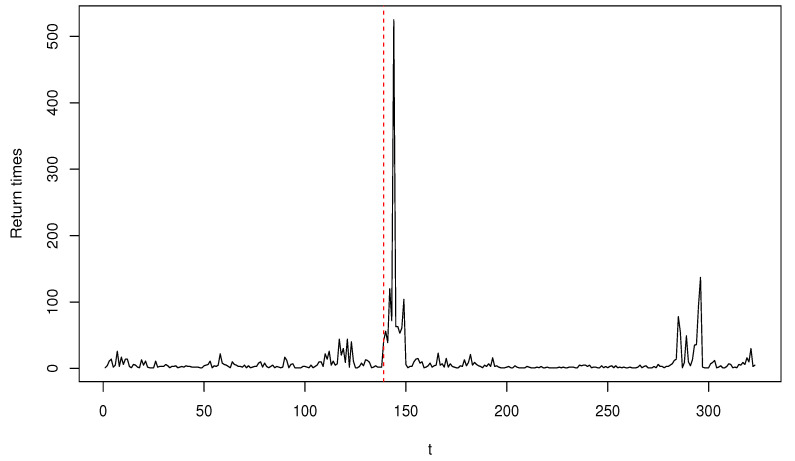
Plot of the return times of extreme events for Goldman Sachs Group (GS) stock.

We first compute the hitting times, $\tau_{1},\tau_{2},\ldots$, for which the log-returns of GS stock fall outside the 0.05 and 0.95 quantiles of the data. The return times of these extreme events are calculated as $Y_{t}=\tau_{t}-\tau_{t-1}$. Figure 1 plots $Y_{t}$, t=1,…,323. The figure shows that the data include large observations; for example, a sample variance of 1106 with a sample mean of 10.01 indicates the existence of aberrant observations.

Since $Y_{t}\geq 1$, we consider a geometric distribution that counts the total number of trials, rather than the number of failures, to fit the following geometric INGARCH(1,1) models to the data:

$$\begin{array}{c} Y_{t}|F_{t-1}∼Geo\left(p_{t}\right),\;\;\;X_{t}=\frac{1}{p_{t}}=d+aX_{t-1}+bY_{t-1}, \end{array}$$

where ${\tilde{X}}_{1}$ is set as the sample mean of the data. Kim and Lee [22] showed that the optimal α for the MDPDE is 0.25, using the criterion provided in Remark 1. The results for the parameter estimation are summarized in Table 11 for α=0 (CMLE) and 0.25 (MDPDE with optimal α); figures in parentheses denote the standard errors of the corresponding estimates. We observe that, compared with the CMLE, the MDPDE with α=0.25 is quite different and has smaller standard errors.

**Figure 2:**
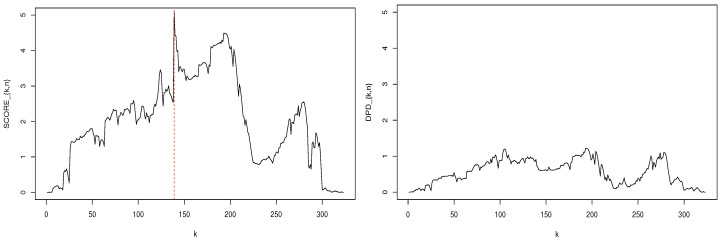
Plots of $SCORE_{k,n}$ and $DPD_{k,n}$.

Next, we use ${\hat{T}}_{n}^{score}$ and ${\hat{T}}_{n}^{0.25}$ (${\hat{T}}_{n}^{\alpha }$ with α=0.25) to perform a parameter change test at the nominal level of 0.05 (the corresponding critical value is 3.004). Let ${\hat{T}}_{n}^{score}=\max_{1\leq k\leq n}SCORE_{k,n}$ and ${\hat{T}}_{n}^{0.25}=\max_{1\leq k\leq n}DPD_{k,n}$. The left and right panels of Figure 2 display $SCORE_{k,n}$ and $DPD_{k,n}$, respectively. For most *k*, $DPD_{k,n}$ appears to be smaller than $SCORE_{k,n}$, which is definitely attributed to the robustness of the MDPDE and DPD. We obtain ${\hat{T}}_{n}^{score}=5.136$, which suggests the existence of a parameter change. In Figure 1 and Figure 2, the red, vertical, dashed line represents the location of a change when ${\hat{T}}_{n}^{score}$ is applied. However, this result is not so reliable because ${\hat{T}}_{n}^{score}$ can signal a change point affected by outliers as seen in the previous section, and the change point is truly detected at the occurrence time of an outlier in this case. In contrast, ${\hat{T}}_{n}^{0.25}$ yields a value of 1.219, indicating that no change point exists. This result clearly demonstrates that outliers can severely affect parameter estimates and change point tests by mistakenly identifying a change point. Our findings confirm that the DPD-based change point test provides a functional and robust alternative to the score-based CUSUM test in the presence of outliers.

## 4. Conclusions

In this study, we developed a DPD-based robust change point test for general integer-valued time series models with a conditional distribution that belongs to the one-parameter exponential family. We provided regularity conditions under which the proposed test converges weakly to the function of a Brownian bridge. The simulation study showed that the DPD-based test produces reasonable sizes and powers regardless of the existence of outliers, whereas the score-based CUSUM test suffers from severe size distortions when the data are contaminated by outliers. In the real data analysis using the return times of extreme events related to GS stock, the score-based CUSUM test supported the existence a parameter change, due to the influence of outliers, while the DPD-based test did not detect a change point because of its robust property. This result confirms the validity of the proposed test as a robust test in practice. It is noteworthy that the DPD-based test can be feasibly extended to other parametric models as far as the asymptotic properties of the MDPDE for the models are validated. We leave the issue of extension to other models as our future study.

## Figures and Tables

**Table 1:** Empirical sizes and powers for Poisson integer-valued generalized autoregressive conditional heteroscedastic (INGARCH)(1,1) models when no outliers exist.

				${\hat{T}}_{n}^{\alpha }$ with α				
	θ=(d,a,b)	n	${\hat{T}}_{n}^{score}$	α=0.1	α=0.2	α=0.3	α=0.5	α=1
	(1, 0.2, 0.2)	500	0.084	0.053	0.059	0.059	0.058	0.059
		1000	0.065	0.047	0.053	0.053	0.051	0.059
Sizes	(1, 0.2, 0.4)	500	0.049	0.040	0.043	0.045	0.047	0.047
		1000	0.033	0.039	0.045	0.047	0.050	0.053
	(1, 0.2, 0.7)	500	0.031	0.028	0.030	0.029	0.029	0.034
		1000	0.050	0.051	0.047	0.044	0.046	0.051
	$\theta^{\prime }=\left(d^{\prime },a^{\prime },b^{\prime }\right)$	**n**	θ=(d,a,b)=(1,0.2,0.2) **changes to** $\theta^{\prime }=\left(d^{\prime },a^{\prime },b^{\prime }\right)$					
	(1.5, 0.2, 0.2)	500	0.836	0.776	0.764	0.741	0.687	0.525
		1000	0.912	0.914	0.911	0.910	0.901	0.871
Powers	(1, 0.4, 0.2)	500	0.782	0.704	0.695	0.661	0.591	0.454
		1000	0.951	0.942	0.939	0.937	0.917	0.886
	(1, 0.2, 0.4)	500	0.819	0.804	0.800	0.795	0.736	0.634
		1000	0.996	0.996	0.996	0.993	0.991	0.978

**Table 2:** Empirical sizes and powers for Poisson INGARCH(1,1) models when p=0.01 and γ=5.

				${\hat{T}}_{n}^{\alpha }$ with α				
	θ=(d,a,b)	n	${\hat{T}}_{n}^{score}$	α=0.1	α=0.2	α=0.3	α=0.5	α=1
	(1, 0.2, 0.2)	500	0.108	0.048	0.046	0.046	0.052	0.057
		1000	0.110	0.048	0.044	0.041	0.050	0.053
Sizes	(1, 0.2, 0.4)	500	0.070	0.041	0.041	0.046	0.046	0.049
		1000	0.078	0.041	0.042	0.041	0.045	0.043
	(1, 0.2, 0.7)	500	0.057	0.035	0.039	0.038	0.045	0.045
		1000	0.061	0.041	0.042	0.045	0.044	0.049
	$\theta^{\prime }=\left(d^{\prime },a^{\prime },b^{\prime }\right)$	**n**	θ=(d,a,b)=(1,0.2,0.2) **changes to** $\theta^{\prime }=\left(d^{\prime },a^{\prime },b^{\prime }\right)$					
	(1.5, 0.2, 0.2)	500	0.792	0.736	0.735	0.723	0.676	0.569
		1000	0.901	0.898	0.903	0.903	0.896	0.856
Powers	(1, 0.4, 0.2)	500	0.766	0.684	0.686	0.667	0.626	0.525
		1000	0.944	0.934	0.935	0.931	0.915	0.864
	(1, 0.2, 0.4)	500	0.871	0.806	0.804	0.787	0.752	0.647
		1000	0.997	0.993	0.993	0.992	0.990	0.960

**Table 3:** Empirical sizes and powers for Poisson INGARCH(1,1) models when p=0.01 and γ=10.

				${\hat{T}}_{n}^{\alpha }$ with α				
	θ=(d,a,b)	n	${\hat{T}}_{n}^{score}$	α=0.1	α=0.2	α=0.3	α=0.5	α=1
	(1, 0.2, 0.2)	500	0.246	0.069	0.075	0.070	0.069	0.079
		1000	0.317	0.071	0.062	0.070	0.070	0.062
Sizes	(1, 0.2, 0.4)	500	0.234	0.053	0.060	0.061	0.052	0.051
		1000	0.262	0.059	0.070	0.072	0.071	0.060
	(1, 0.2, 0.7)	500	0.127	0.040	0.040	0.037	0.041	0.038
		1000	0.115	0.045	0.044	0.049	0.048	0.050
	$\theta^{\prime }=\left(d^{\prime },a^{\prime },b^{\prime }\right)$	**n**	θ=(d,a,b)=(1,0.2,0.2) **changes to** $\theta^{\prime }=\left(d^{\prime },a^{\prime },b^{\prime }\right)$					
	(1.5, 0.2, 0.2)	500	0.840	0.785	0.791	0.769	0.742	0.649
		1000	0.874	0.863	0.874	0.869	0.868	0.862
Powers	(1, 0.4, 0.2)	500	0.835	0.743	0.759	0.740	0.694	0.590
		1000	0.911	0.910	0.913	0.908	0.902	0.879
	(1, 0.2, 0.4)	500	0.920	0.829	0.835	0.831	0.787	0.697
		1000	0.997	0.992	0.995	0.997	0.994	0.965

**Table 4:** Empirical sizes and powers for Poisson INGARCH(1,1) models when p=0.03 and γ=5.

				${\hat{T}}_{n}^{\alpha }$ with α				
	θ=(d,a,b)	n	${\hat{T}}_{n}^{score}$	α=0.1	α=0.2	α=0.3	α=0.5	α=1
	(1, 0.2, 0.2)	500	0.213	0.060	0.059	0.058	0.062	0.074
		1000	0.229	0.052	0.055	0.063	0.062	0.061
Sizes	(1, 0.2, 0.4)	500	0.176	0.052	0.057	0.064	0.066	0.060
		1000	0.173	0.047	0.055	0.054	0.055	0.059
	(1, 0.2, 0.7)	500	0.073	0.030	0.039	0.037	0.037	0.045
		1000	0.086	0.039	0.035	0.040	0.042	0.039
	$\theta^{\prime }=\left(d^{\prime },a^{\prime },b^{\prime }\right)$	**n**	θ=(d,a,b)=(1,0.2,0.2) **changes to** $\theta^{\prime }=\left(d^{\prime },a^{\prime },b^{\prime }\right)$					
	(1.5, 0.2, 0.2)	500	0.804	0.693	0.715	0.709	0.687	0.616
		1000	0.867	0.859	0.867	0.867	0.859	0.847
Powers	(1, 0.4, 0.2)	500	0.786	0.662	0.693	0.681	0.634	0.561
		1000	0.908	0.896	0.903	0.899	0.893	0.868
	(1, 0.2, 0.4)	500	0.915	0.787	0.797	0.792	0.773	0.672
		1000	0.998	0.994	0.995	0.993	0.986	0.965

**Table 5:** Empirical sizes and powers for Poisson INGARCH(1,1) models when p=0.03 and γ=10.

				${\hat{T}}_{n}^{\alpha }$ with α				
	θ=(d,a,b)	n	${\hat{T}}_{n}^{score}$	α=0.1	α=0.2	α=0.3	α=0.5	α=1
	(1, 0.2, 0.2)	500	0.475	0.083	0.082	0.083	0.091	0.102
		1000	0.592	0.092	0.097	0.104	0.109	0.097
Sizes	(1, 0.2, 0.4)	500	0.556	0.071	0.078	0.080	0.068	0.065
		1000	0.621	0.092	0.113	0.115	0.108	0.071
	(1, 0.2, 0.7)	500	0.296	0.050	0.056	0.056	0.053	0.040
		1000	0.289	0.060	0.062	0.057	0.060	0.055
	$\theta^{\prime }=\left(d^{\prime },a^{\prime },b^{\prime }\right)$	**n**	θ=(d,a,b)=(1,0.2,0.2) **changes to** $\theta^{\prime }=\left(d^{\prime },a^{\prime },b^{\prime }\right)$					
	(1.5, 0.2, 0.2)	500	0.834	0.760	0.800	0.801	0.782	0.719
		1000	0.889	0.821	0.852	0.867	0.860	0.866
Powers	(1, 0.4, 0.2)	500	0.850	0.738	0.783	0.786	0.759	0.688
		1000	0.897	0.848	0.887	0.889	0.895	0.880
	(1, 0.2, 0.4)	500	0.951	0.817	0.847	0.842	0.815	0.728
		1000	0.997	0.991	0.992	0.992	0.983	0.969

**Table 6:** Empirical sizes and powers for negative binomial INGARCH (NB-INGARCH)(1,1) models when no outliers exist.

				${\hat{T}}_{n}^{\alpha }$ with α				
	θ=(d,a,b)	n	${\hat{T}}_{n}^{score}$	α=0.1	α=0.2	α=0.3	α=0.5	α=1
	(1, 0.2, 0.2)	500	0.076	0.050	0.052	0.054	0.061	0.071
		1000	0.061	0.055	0.052	0.052	0.055	0.059
Sizes	(1, 0.2, 0.4)	500	0.040	0.041	0.038	0.040	0.045	0.048
		1000	0.049	0.053	0.056	0.057	0.062	0.060
	(1, 0.2, 0.7)	500	0.047	0.046	0.043	0.038	0.042	0.043
		1000	0.041	0.044	0.048	0.048	0.047	0.043
	$\theta^{\prime }=\left(d^{\prime },a^{\prime },b^{\prime }\right)$	**n**	θ=(d,a,b)=(1,0.2,0.2) **changes to** $\theta^{\prime }=\left(d^{\prime },a^{\prime },b^{\prime }\right)$					
	(1.5, 0.2, 0.2)	500	0.821	0.759	0.735	0.706	0.640	0.505
		1000	0.953	0.942	0.936	0.932	0.919	0.881
Powers	(1, 0.4, 0.2)	500	0.759	0.689	0.646	0.611	0.558	0.454
		1000	0.967	0.964	0.959	0.955	0.940	0.881
	(1, 0.2, 0.4)	500	0.733	0.719	0.718	0.702	0.650	0.544
		1000	0.984	0.984	0.981	0.975	0.961	0.908

**Table 7:** Empirical sizes and powers for NB-INGARCH(1,1) models when p=0.01 and κ=0.6.

				${\hat{T}}_{n}^{\alpha }$ with α				
	θ=(d,a,b)	n	${\hat{T}}_{n}^{score}$	α=0.1	α=0.2	α=0.3	α=0.5	α=1
	(1, 0.2, 0.2)	500	0.158	0.062	0.066	0.066	0.071	0.071
		1000	0.173	0.069	0.066	0.067	0.068	0.061
Sizes	(1, 0.2, 0.4)	500	0.105	0.045	0.045	0.049	0.047	0.039
		1000	0.112	0.058	0.058	0.062	0.057	0.047
	(1, 0.2, 0.7)	500	0.045	0.031	0.035	0.038	0.041	0.038
		1000	0.065	0.042	0.045	0.044	0.041	0.045
	$\theta^{\prime }=\left(d^{\prime },a^{\prime },b^{\prime }\right)$	**n**	θ=(d,a,b)=(1,0.2,0.2) **changes to** $\theta^{\prime }=\left(d^{\prime },a^{\prime },b^{\prime }\right)$					
	(1.5, 0.2, 0.2)	500	0.803	0.705	0.714	0.695	0.647	0.516
		1000	0.945	0.931	0.931	0.930	0.921	0.909
Powers	(1, 0.4, 0.2)	500	0.757	0.648	0.645	0.626	0.579	0.464
		1000	0.959	0.958	0.952	0.947	0.930	0.895
	(1, 0.2, 0.4)	500	0.807	0.704	0.716	0.710	0.659	0.574
		1000	0.985	0.978	0.980	0.979	0.969	0.935

**Table 8:** Empirical sizes and powers for NB-INGARCH(1,1) models when p=0.01 and κ=0.5.

				${\hat{T}}_{n}^{\alpha }$ with α				
	θ=(d,a,b)	n	${\hat{T}}_{n}^{score}$	α=0.1	α=0.2	α=0.3	α=0.5	α=1
	(1, 0.2, 0.2)	500	0.258	0.069	0.069	0.070	0.076	0.080
		1000	0.292	0.061	0.061	0.057	0.058	0.068
Sizes	(1, 0.2, 0.4)	500	0.177	0.048	0.048	0.052	0.057	0.058
		1000	0.236	0.072	0.079	0.081	0.073	0.074
	(1, 0.2, 0.7)	500	0.095	0.048	0.054	0.058	0.060	0.055
		1000	0.097	0.049	0.050	0.050	0.050	0.051
	$\theta^{\prime }=\left(d^{\prime },a^{\prime },b^{\prime }\right)$	**n**	θ=(d,a,b)=(1,0.2,0.2) **changes to** $\theta^{\prime }=\left(d^{\prime },a^{\prime },b^{\prime }\right)$					
	(1.5, 0.2, 0.2)	500	0.840	0.771	0.768	0.740	0.688	0.599
		1000	0.923	0.924	0.932	0.926	0.925	0.897
Powers	(1, 0.4, 0.2)	500	0.808	0.704	0.709	0.673	0.634	0.536
		1000	0.938	0.946	0.946	0.943	0.935	0.898
	(1, 0.2, 0.4)	500	0.842	0.723	0.740	0.735	0.696	0.586
		1000	0.997	0.989	0.984	0.977	0.972	0.923

**Table 9:** Empirical sizes and powers for NB-INGARCH(1,1) models when p=0.03 and κ=0.6.

				${\hat{T}}_{n}^{\alpha }$ with α				
	θ=(d,a,b)	n	${\hat{T}}_{n}^{score}$	α=0.1	α=0.2	α=0.3	α=0.5	α=1
	(1, 0.2, 0.2)	500	0.289	0.079	0.077	0.076	0.086	0.069
		1000	0.328	0.060	0.068	0.068	0.077	0.075
Sizes	(1, 0.2, 0.4)	500	0.228	0.051	0.054	0.051	0.052	0.047
		1000	0.246	0.054	0.064	0.066	0.064	0.059
	(1, 0.2, 0.7)	500	0.090	0.035	0.040	0.040	0.044	0.036
		1000	0.108	0.058	0.053	0.052	0.050	0.040
	$\theta^{\prime }=\left(d^{\prime },a^{\prime },b^{\prime }\right)$	**n**	θ=(d,a,b)=(1,0.2,0.2) **changes to** $\theta^{\prime }=\left(d^{\prime },a^{\prime },b^{\prime }\right)$					
	(1.5, 0.2, 0.2)	500	0.818	0.685	0.705	0.702	0.675	0.582
		1000	0.925	0.892	0.900	0.899	0.905	0.909
Powers	(1, 0.4, 0.2)	500	0.806	0.637	0.666	0.664	0.627	0.522
		1000	0.938	0.927	0.926	0.922	0.913	0.896
	(1, 0.2, 0.4)	500	0.870	0.690	0.734	0.731	0.704	0.604
		1000	0.990	0.976	0.978	0.974	0.969	0.931

**Table 10:** Empirical sizes and powers for NB-INGARCH(1,1) models when p=0.03 and κ=0.5.

				${\hat{T}}_{n}^{\alpha }$ with α				
	θ=(d,a,b)	n	${\hat{T}}_{n}^{score}$	α=0.1	α=0.2	α=0.3	α=0.5	α=1
	(1, 0.2, 0.2)	500	0.469	0.085	0.088	0.100	0.102	0.096
		1000	0.563	0.075	0.088	0.097	0.105	0.100
Sizes	(1, 0.2, 0.4)	500	0.506	0.068	0.071	0.076	0.081	0.072
		1000	0.532	0.089	0.096	0.101	0.089	0.078
	(1, 0.2, 0.7)	500	0.188	0.054	0.066	0.072	0.066	0.061
		1000	0.207	0.053	0.051	0.064	0.069	0.059
	$\theta^{\prime }=\left(d^{\prime },a^{\prime },b^{\prime }\right)$	**n**	θ=(d,a,b)=(1,0.2,0.2) **changes to** $\theta^{\prime }=\left(d^{\prime },a^{\prime },b^{\prime }\right)$					
	(1.5, 0.2, 0.2)	500	0.879	0.749	0.784	0.797	0.758	0.687
		1000	0.930	0.880	0.889	0.893	0.889	0.886
Powers	(1, 0.4, 0.2)	500	0.867	0.698	0.766	0.756	0.734	0.636
		1000	0.948	0.891	0.900	0.906	0.906	0.889
	(1, 0.2, 0.4)	500	0.927	0.735	0.770	0.770	0.743	0.639
		1000	0.995	0.977	0.984	0.981	0.971	0.944

**Table 11:** Parameter estimates for geometric INGARCH(1,1) models.

α	$\hat{d}$	$\hat{a}$	$\hat{b}$	$\hat{\mathrm{AMSE}}$
0(CMLE)	0.526(0.406)	0.490(0.175)	0.483(0.156)	0.623
0.25	0.432(0.242)	0.518(0.129)	0.418(0.115)	0.398

## Appendix A

In this appendix, we prove Theorem 1 for α>0; refer to Lee and Lee [14] for the case of α=0. The following properties of the probability mass function of the non-negative integer-valued exponential family are useful for proving Lemma A1. For all $y\in N_{0}$ and η∈R:

(E1) 0<p(y|η)<1,
(E2) $\sum_{y=0}^{\infty }p\left(y|\eta \right)=1$,
(E3) $\sum_{y=0}^{\infty }yp\left(y|\eta \right)=B\left(\eta \right)$,
(E4) $\sum_{y=0}^{\infty }y^{2}p\left(y|\eta \right)=B^{\prime }\left(\eta \right)+B{\left(\eta \right)}^{2}$,
(E5) $\sum_{y=0}^{\infty }y^{3}p\left(y|\eta \right)=B^{\prime \prime }\left(\eta \right)+3B^{\prime }\left(\eta \right)B\left(\eta \right)+B{\left(\eta \right)}^{3}$.

Throughout this section, we denote $L_{\alpha ,n}\left(\theta \right)=n^{-1}\sum_{t=1}^{n}l_{\alpha ,t}\left(\theta \right)$ and employ the notation $\eta_{t}=\eta_{t}\left(\theta \right),\;{\tilde{\eta }}_{t}={\tilde{\eta }}_{t}\left(\theta \right)$, and $\eta_{t}^{0}=\eta_{t}\left(\theta_{0}\right)$ for brevity. Furthermore, if we define two functions $h_{\alpha }\left(\eta \right)$ and $m_{\alpha }\left(\eta \right)$ as

$$\begin{array}{cc} h_{\alpha }\left(\eta \right) & =\sum_{y=0}^{\infty }p{\left(y|\eta \right)}^{1+\alpha }\frac{y-B(\eta )}{B^{\prime }\left(\eta \right)}-p{\left(Y_{t}|\eta \right)}^{\alpha }\frac{Y_{t}-B\left(\eta \right)}{B^{\prime }\left(\eta \right)},\\ m_{\alpha }\left(\eta \right) & =\sum_{y=0}^{\infty }p{\left(y|\eta \right)}^{1+\alpha }\left[\left(1+\alpha \right){\left(\frac{y-B(\eta )}{B^{\prime }\left(\eta \right)}\right)}^{2}-\frac{B^{\prime \prime }\left(\eta \right)}{B^{\prime }{\left(\eta \right)}^{2}}\frac{y-B(\eta )}{B^{\prime }\left(\eta \right)}-\frac{1}{B^{\prime }\left(\eta \right)}\right]\\ & -p{\left(Y_{t}|\eta \right)}^{\alpha }\left[\alpha {\left(\frac{Y_{t}-B\left(\eta \right)}{B^{\prime }\left(\eta \right)}\right)}^{2}-\frac{B^{\prime \prime }\left(\eta \right)}{B^{\prime }{\left(\eta \right)}^{2}}\frac{Y_{t}-B\left(\eta \right)}{B^{\prime }\left(\eta \right)}-\frac{1}{B^{\prime }\left(\eta \right)}\right], \end{array}$$

we obtain

$$\begin{array}{ccc} \frac{\partial l_{\alpha ,t}\left(\theta \right)}{\partial \theta } & = & \left(1+\alpha \right)h_{\alpha }\left(\eta_{t}\right)\frac{\partial X_{t}\left(\theta \right)}{\partial \theta },\\ \frac{\partial^{2}l_{\alpha ,t}\left(\theta \right)}{\partial \theta \partial \theta^{T}} & = & \left(1+\alpha \right)\left(h_{\alpha }\left(\eta_{t}\right)\frac{\partial^{2}X_{t}\left(\theta \right)}{\partial \theta \partial \theta^{T}}+m_{\alpha }\left(\eta_{t}\right)\frac{\partial X_{t}\left(\theta \right)}{\partial \theta }\frac{\partial X_{t}\left(\theta \right)}{\partial \theta^{T}}\right). \end{array}$$

**Lemma** **A1.**
*Suppose that conditions* **(A3)** *,* **(A6)** *, and* **(A11)** *hold. Then, we have*

$$\begin{array}{c} |h_{\alpha }\left(\eta_{t}\right)|\leq \frac{1}{\underline{c}}\left(Y_{t}+3X_{t}\left(\theta \right)\right),\\ |h_{\alpha }\left({\tilde{\eta }}_{t}\right)|\leq \frac{1}{\underline{c}}(Y_{t}+3X_{t}\left(\theta \right)+3|X_{t}\left(\theta \right)-{\tilde{X}}_{t}\left(\theta \right)\left|\right),\\ |m_{\alpha }\left(\eta_{t}\right)|\leq \frac{\alpha }{{\underline{c}}^{2}}Y_{t}^{2}+\frac{K}{{\underline{c}}^{1/2}}Y_{t}+\frac{\alpha }{{\underline{c}}^{2}}X_{t}{\left(\theta \right)}^{2}+\frac{3K}{{\underline{c}}^{1/2}}X_{t}\left(\theta \right)+\frac{3+\alpha }{\underline{c}},\\ |h_{\alpha }\left(\eta_{t}\right)-h_{\alpha }\left({\tilde{\eta }}_{t}\right)|\leq \left[\frac{\alpha }{{\underline{c}}^{2}}Y_{t}^{2}+\frac{K}{{\underline{c}}^{1/2}}Y_{t}+\frac{2\alpha }{{\underline{c}}^{2}}\left(X_{t}{\left(\theta \right)}^{2}+{\left|X_{t}\left(\theta \right)-{\tilde{X}}_{t}\left(\theta \right)\right|}^{2}\right)\right.\\ \;\;\;\;\;\;\;\;\;\;\;\;\;\;\;\;\;\;\;\;\;\;\;\;\;\;\;\;\;\;\;\;\;\;\;\;\left.+\frac{3K}{{\underline{c}}^{1/2}}\left(X_{t}\left(\theta \right)+\left|X_{t}\left(\theta \right)-{\tilde{X}}_{t}\left(\theta \right)\right|\right)+\frac{3+\alpha }{\underline{c}}\right]\left|X_{t}\left(\theta \right)-{\tilde{X}}_{t}\left(\theta \right)\right|,\\ |m_{\alpha }\left(\tilde{\eta_{t}}\right)|\leq \frac{\alpha }{{\underline{c}}^{2}}Y_{t}^{2}+\frac{K}{{\underline{c}}^{1/2}}Y_{t}+\frac{2\alpha }{{\underline{c}}^{2}}\left(X_{t}{\left(\theta \right)}^{2}+{\left|X_{t}\left(\theta \right)-{\tilde{X}}_{t}\left(\theta \right)\right|}^{2}\right)\\ \;\;\;\;\;\;\;\;\;\;\;\;\;\;\;\;\;\;\;+\frac{3K}{{\underline{c}}^{1/2}}\left(X_{t}\left(\theta \right)+\left|X_{t}\left(\theta \right)-{\tilde{X}}_{t}\left(\theta \right)\right|\right)+\frac{3+\alpha }{\underline{c}},\\ |m_{\alpha }\left(\eta_{t}\right)-m_{\alpha }\left({\tilde{\eta }}_{t}\right)|\leq \left[\frac{\alpha^{2}}{{\underline{c}}^{3}}Y_{t}^{3}+\frac{3\alpha K}{{\underline{c}}^{3/2}}Y_{t}^{2}+\left(\frac{3\alpha }{{\underline{c}}^{2}}+M+3K^{2}\right)Y_{t}\right.\\ \;\;\;\;\;\;\;\;\;\;\;\;\;\;\;\;\;\;\;\;\;\;\;\;\;\;\;\;\;\;\;\;\;\;\;\;\;\;\left.+\frac{4(3\alpha^{2}+4\alpha +2)}{{\underline{c}}^{3}}\left(X_{t}{\left(\theta \right)}^{3}+{\left|X_{t}\left(\theta \right)-{\tilde{X}}_{t}\left(\theta \right)\right|}^{3}\right)\right.\\ \;\;\;\;\;\;\;\;\;\;\;\;\;\;\;\;\;\;\;\;\;\;\;\;\;\;\;\;\;\;\;\;\;\;\;\;\;\;\left.+\frac{6\alpha K}{{\underline{c}}^{3/2}}\left(X_{t}{\left(\theta \right)}^{2}+{\left|X_{t}\left(\theta \right)-{\tilde{X}}_{t}\left(\theta \right)\right|}^{2}\right)\right.\\ \;\;\;\;\;\;\;\;\;\;\;\;\;\;\;\;\;\;\;\;\;\;\;\;\;\;\;\;\;\;\;\;\;\;\;\;\;\;\left.+3\left(\frac{\alpha^{2}+5\alpha +3}{{\underline{c}}^{2}}+M+3K^{2}\right)\left(X_{t}\left(\theta \right)+\left|X_{t}\left(\theta \right)-{\tilde{X}}_{t}\left(\theta \right)\right|\right)\right.\\ \;\;\;\;\;\;\;\;\;\;\;\;\;\;\;\;\;\;\;\;\;\;\;\;\;\;\;\;\;\;\;\;\;\;\;\;\;\;\left.+\frac{(\alpha^{2}+5\alpha +8)K}{{\underline{c}}^{1/2}}\right]\left|X_{t}\left(\theta \right)-{\tilde{X}}_{t}\left(\theta \right)\right|. \end{array}$$

**Proof.** The proofs for the first four parts of the lemma can be found in Lemma 4 of Kim and Lee [22]. The fifth part is obtained directly from the third part, together with the fact that ${\tilde{X}}_{t}\left(\theta \right)\leq \left|X_{t}\left(\theta \right)-{\tilde{X}}_{t}\left(\theta \right)\right|+X_{t}\left(\theta \right)$. By the MVT, (E1)–(E5), **(A3)**, **(A6)**, and **(A11)**, we have

$$\begin{array}{cc} & |m_{\alpha }\left(\eta_{t}\right)-m_{\alpha }\left({\tilde{\eta }}_{t}\right)|\\ = & \left|\frac{\partial m_{\alpha }\left(B^{-1}\left(X_{t}^{*}\left(\theta \right)\right)\right)}{\partial X_{t}\left(\theta \right)}\right|\left|X_{t}\left(\theta \right)-{\tilde{X}}_{t}\left(\theta \right)\right|\\ = & \left|\frac{\partial m_{\alpha }\left(\eta_{t}^{*}\right)}{\partial \eta_{t}}\frac{1}{B^{\prime }\left(\eta_{t}^{*}\right)}\right|\left|X_{t}\left(\theta \right)-{\tilde{X}}_{t}\left(\theta \right)\right|\\ = & \frac{1}{B^{\prime }\left(\eta_{t}^{*}\right)}\left|\sum_{y=0}^{\infty }p{\left(y|\eta_{t}^{*}\right)}^{1+\alpha }\left[{\left(1+\alpha \right)}^{2}\frac{1}{B^{\prime }{\left(\eta_{t}^{*}\right)}^{2}}{\left(y-B\left(\eta_{t}^{*}\right)\right)}^{3}-3\left(1+\alpha \right)\frac{B^{\prime \prime }\left(\eta_{t}^{*}\right)}{B^{\prime }{\left(\eta_{t}^{*}\right)}^{3}}{\left(y-B\left(\eta_{t}^{*}\right)\right)}^{2}\right.\right.\\ & \;\;\;\;\;\;\;\;\;\;\;\;\;\;\left.\left.+\left(-3\left(1+\alpha \right)\frac{1}{B^{\prime }\left(\eta_{t}^{*}\right)}-\frac{B^{\left(3\right)}\left(\eta_{t}^{*}\right)}{B^{\prime }{\left(\eta_{t}^{*}\right)}^{3}}+3\frac{B^{\prime \prime }{\left(\eta_{t}^{*}\right)}^{2}}{B^{\prime }{\left(\eta_{t}^{*}\right)}^{4}}\right)\left(y-B\left(\eta_{t}^{*}\right)\right)+2\frac{B^{\prime \prime }\left(\eta_{t}^{*}\right)}{B^{\prime }{\left(\eta_{t}^{*}\right)}^{2}}\right]\right.\\ & \;\;\;\;\;\;\;\;\;\;\;\;\;\;\left.-p{\left(Y_{t}|\eta_{t}^{*}\right)}^{\alpha }\left[\alpha^{2}\frac{1}{B^{\prime }{\left(\eta_{t}^{*}\right)}^{2}}{\left(Y_{t}-B\left(\eta_{t}^{*}\right)\right)}^{3}-3\alpha \frac{B^{\prime \prime }\left(\eta_{t}^{*}\right)}{B^{\prime }{\left(\eta_{t}^{*}\right)}^{3}}{\left(Y_{t}-B\left(\eta_{t}^{*}\right)\right)}^{2}\right.\right.\\ & \;\;\;\;\;\;\;\;\;\;\;\;\;\;\left.\left.+\left(-3\alpha \frac{1}{B^{\prime }\left(\eta_{t}^{*}\right)}-\frac{B^{\left(3\right)}\left(\eta_{t}^{*}\right)}{B^{\prime }{\left(\eta_{t}^{*}\right)}^{3}}+3\frac{B^{\prime \prime }{\left(\eta_{t}^{*}\right)}^{2}}{B^{\prime }{\left(\eta_{t}^{*}\right)}^{4}}\right)\left(Y_{t}-B\left(\eta_{t}^{*}\right)\right)+2\frac{B^{\prime \prime }\left(\eta_{t}^{*}\right)}{B^{\prime }{\left(\eta_{t}^{*}\right)}^{2}}\right]\right|\left|X_{t}\left(\theta \right)-{\tilde{X}}_{t}\left(\theta \right)\right|\\ \leq & \left[{\left(1+\alpha \right)}^{2}\frac{1}{B^{\prime }{\left(\eta_{t}^{*}\right)}^{3}}\left(B^{\prime \prime }\left(\eta_{t}^{*}\right)+3B^{\prime }\left(\eta_{t}^{*}\right)B\left(\eta_{t}^{*}\right)+B{\left(\eta_{t}^{*}\right)}^{3}+B{\left(\eta_{t}^{*}\right)}^{3}\right)+3\left(1+\alpha \right)\left|\frac{B^{\prime \prime }\left(\eta_{t}^{*}\right)}{B^{\prime }{\left(\eta_{t}^{*}\right)}^{3}}\right|\right.\\ & \;\;\left.+\left(3\left(1+\alpha \right)\frac{1}{B^{\prime }{\left(\eta_{t}^{*}\right)}^{2}}+\left|\frac{B^{\left(3\right)}\left(\eta_{t}^{*}\right)}{B^{\prime }{\left(\eta_{t}^{*}\right)}^{4}}\right|+3\frac{B^{\prime \prime }{\left(\eta_{t}^{*}\right)}^{2}}{B^{\prime }{\left(\eta_{t}^{*}\right)}^{5}}\right)\left(B\left(\eta_{t}^{*}\right)+B\left(\eta_{t}^{*}\right)\right)+2\left|\frac{B^{\prime \prime }\left(\eta_{t}^{*}\right)}{B^{\prime }{\left(\eta_{t}^{*}\right)}^{3}}\right|\right.\\ & \;\;\left.+\alpha^{2}\frac{1}{B^{\prime }{\left(\eta_{t}^{*}\right)}^{3}}\left(Y_{t}^{3}+B{\left(\eta_{t}^{*}\right)}^{3}\right)+3\alpha \left|\frac{B^{\prime \prime }\left(\eta_{t}^{*}\right)}{B^{\prime }{\left(\eta_{t}^{*}\right)}^{4}}\right|\left(Y_{t}^{2}+B{\left(\eta_{t}^{*}\right)}^{2}\right)\right.\\ & \;\;\left.+\left(3\alpha \frac{1}{B^{\prime }{\left(\eta_{t}^{*}\right)}^{2}}+\left|\frac{B^{\left(3\right)}\left(\eta_{t}^{*}\right)}{B^{\prime }{\left(\eta_{t}^{*}\right)}^{4}}\right|+3\frac{B^{\prime \prime }{\left(\eta_{t}^{*}\right)}^{2}}{B^{\prime }{\left(\eta_{t}^{*}\right)}^{5}}\right)\left(Y_{t}+B\left(\eta_{t}^{*}\right)\right)+2\left|\frac{B^{\prime \prime }\left(\eta_{t}^{*}\right)}{B^{\prime }{\left(\eta_{t}^{*}\right)}^{3}}\right|\right]\left|X_{t}\left(\theta \right)-{\tilde{X}}_{t}\left(\theta \right)\right|\\ \leq & \left[\frac{\alpha^{2}}{{\underline{c}}^{3}}Y_{t}^{3}+\frac{3\alpha K}{{\underline{c}}^{3/2}}Y_{t}^{2}+\left(\frac{3\alpha }{{\underline{c}}^{2}}+M+3K^{2}\right)Y_{t}+\frac{3\alpha^{2}+4\alpha +2}{{\underline{c}}^{3}}B{\left(\eta_{t}^{*}\right)}^{3}+\frac{3\alpha K}{{\underline{c}}^{3/2}}B{\left(\eta_{t}^{*}\right)}^{2}\right.\\ & \;\;\left.+\left(\frac{3\alpha^{2}+15\alpha +9}{{\underline{c}}^{2}}+3M+9K^{2}\right)B\left(\eta_{t}^{*}\right)+\frac{(\alpha^{2}+5\alpha +8)K}{{\underline{c}}^{1/2}}\right]\left|X_{t}\left(\theta \right)-{\tilde{X}}_{t}\left(\theta \right)\right|, \end{array}$$

where $X_{t}^{*}\left(\theta \right)$ is an intermediate point between $X_{t}\left(\theta \right)$ and ${\tilde{X}}_{t}\left(\theta \right)$, and $\eta_{t}^{*}=B^{-1}\left(X_{t}^{*}\left(\theta \right)\right)$. Note that since $B^{-1}$ is strictly increasing, $\eta_{t}^{*}$ lies between $B^{-1}\left(X_{t}\left(\theta \right)\right)=\eta_{t}$ and $B^{-1}\left({\tilde{X}}_{t}\left(\theta \right)\right)={\tilde{\eta }}_{t}$. Then, because $B\left(\eta_{t}^{*}\right)\leq B\left(\eta_{t}\right)+\left|B\left(\eta_{t}\right)-B\left({\tilde{\eta }}_{t}\right)\right|$, the last part of the lemma is established. □

**Lemma** **A2.**
*Suppose that conditions* **(A0)** *–* **(A11)** *hold. Then, under $H_{0}$, we have as n→∞,*

$$\frac{1}{n}\sum_{t=1}^{n}\underset{\theta \in \Theta }{\sup}∥\frac{\partial^{2}l_{\alpha ,t}\left(\theta \right)}{\partial \theta \partial \theta^{T}}-\frac{\partial^{2}{\tilde{l}}_{\alpha ,t}\left(\theta \right)}{\partial \theta \partial \theta^{T}}∥=o\left(1\right)\;\;a.s.$$

*and*

$$\frac{1}{n}\sum_{t=1}^{n}\underset{\theta \in \Theta }{\sup}∥\frac{\partial l_{\alpha ,t}\left(\theta \right)}{\partial \theta }\frac{\partial l_{\alpha ,t}\left(\theta \right)}{\partial \theta^{T}}-\frac{\partial {\tilde{l}}_{\alpha ,t}\left(\theta \right)}{\partial \theta }\frac{\partial {\tilde{l}}_{\alpha ,t}\left(\theta \right)}{\partial \theta^{T}}∥=o\left(1\right)\;\;a.s.$$

**Proof.** It is sufficient to show that as t→∞,

$$\begin{array}{c} \underset{\theta \in \Theta }{\sup}∥\frac{\partial^{2}l_{\alpha ,t}\left(\theta \right)}{\partial \theta \partial \theta^{T}}-\frac{\partial^{2}{\tilde{l}}_{\alpha ,t}\left(\theta \right)}{\partial \theta \partial \theta^{T}}∥=o\left(1\right)\;\;a.s. \end{array}$$

and

$$\begin{array}{c} \underset{\theta \in \Theta }{\sup}∥\frac{\partial l_{\alpha ,t}\left(\theta \right)}{\partial \theta }\frac{\partial l_{\alpha ,t}\left(\theta \right)}{\partial \theta^{T}}-\frac{\partial {\tilde{l}}_{\alpha ,t}\left(\theta \right)}{\partial \theta }\frac{\partial {\tilde{l}}_{\alpha ,t}\left(\theta \right)}{\partial \theta^{T}}∥=o\left(1\right)\;\;a.s. \end{array}$$

Note that we can write

$$\begin{array}{cc} & \frac{1}{1+\alpha }\underset{\theta \in \Theta }{\sup}∥\frac{\partial^{2}l_{\alpha ,t}\left(\theta \right)}{\partial \theta \partial \theta^{T}}-\frac{\partial^{2}{\tilde{l}}_{\alpha ,t}\left(\theta \right)}{\partial \theta \partial \theta^{T}}∥\\ \leq & \underset{\theta \in \Theta }{\sup}∥h_{\alpha }\left({\tilde{\eta }}_{t}\right)\left(\frac{\partial^{2}X_{t}\left(\theta \right)}{\partial \theta \partial \theta^{T}}-\frac{\partial^{2}{\tilde{X}}_{t}\left(\theta \right)}{\partial \theta \partial \theta^{T}}\right)∥+\underset{\theta \in \Theta }{\sup}∥\left(h_{\alpha }\left(\eta_{t}\right)-h_{\alpha }\left({\tilde{\eta }}_{t}\right)\right)\frac{\partial^{2}X_{t}\left(\theta \right)}{\partial \theta \partial \theta^{T}}∥\\ & +\underset{\theta \in \Theta }{\sup}∥\left(m_{\alpha }\left(\eta_{t}\right)-m_{\alpha }\left({\tilde{\eta }}_{t}\right)\right)\frac{\partial X_{t}\left(\theta \right)}{\partial \theta }\frac{\partial X_{t}\left(\theta \right)}{\partial \theta^{T}}∥+\underset{\theta \in \Theta }{\sup}∥m_{\alpha }\left({\tilde{\eta }}_{t}\right)\frac{\partial X_{t}\left(\theta \right)}{\partial \theta }\left(\frac{\partial X_{t}\left(\theta \right)}{\partial \theta^{T}}-\frac{\partial {\tilde{X}}_{t}\left(\theta \right)}{\partial \theta^{T}}\right)∥\\ & +\underset{\theta \in \Theta }{\sup}∥m_{\alpha }\left({\tilde{\eta }}_{t}\right)\left(\frac{\partial X_{t}\left(\theta \right)}{\partial \theta }-\frac{\partial {\tilde{X}}_{t}\left(\theta \right)}{\partial \theta }\right)\left(\frac{\partial {\tilde{X}}_{t}\left(\theta \right)}{\partial \theta^{T}}-\frac{\partial X_{t}\left(\theta \right)}{\partial \theta^{T}}\right)∥\\ & +\underset{\theta \in \Theta }{\sup}∥m_{\alpha }\left({\tilde{\eta }}_{t}\right)\left(\frac{\partial X_{t}\left(\theta \right)}{\partial \theta }-\frac{\partial {\tilde{X}}_{t}\left(\theta \right)}{\partial \theta }\right)\frac{\partial X_{t}\left(\theta \right)}{\partial \theta^{T}}∥\\ \leq & \underset{\theta \in \Theta }{\sup}|h_{\alpha }\left({\tilde{\eta }}_{t}\right)|\underset{\theta \in \Theta }{\sup}∥\frac{\partial^{2}X_{t}\left(\theta \right)}{\partial \theta \partial \theta^{T}}-\frac{\partial^{2}{\tilde{X}}_{t}\left(\theta \right)}{\partial \theta \partial \theta^{T}}∥+\underset{\theta \in \Theta }{\sup}\left|h_{\alpha }\left(\eta_{t}\right)-h_{\alpha }\left({\tilde{\eta }}_{t}\right)\right|\underset{\theta \in \Theta }{\sup}∥\frac{\partial^{2}X_{t}\left(\theta \right)}{\partial \theta \partial \theta^{T}}∥\\ & +\underset{\theta \in \Theta }{\sup}|m_{\alpha }\left(\eta_{t}\right)-m_{\alpha }\left({\tilde{\eta }}_{t}\right)|{\left(\underset{\theta \in \Theta }{\sup}∥\frac{\partial X_{t}\left(\theta \right)}{\partial \theta }∥\right)}^{2}+2\underset{\theta \in \Theta }{\sup}\left|m_{\alpha }\left({\tilde{\eta }}_{t}\right)\right|\underset{\theta \in \Theta }{\sup}∥\frac{\partial X_{t}\left(\theta \right)}{\partial \theta }∥\underset{\theta \in \Theta }{\sup}∥\frac{\partial X_{t}\left(\theta \right)}{\partial \theta }-\frac{\partial {\tilde{X}}_{t}\left(\theta \right)}{\partial \theta }∥\\ & +\underset{\theta \in \Theta }{\sup}\left|m_{\alpha }\left({\tilde{\eta }}_{t}\right)\right|{\left(\underset{\theta \in \Theta }{\sup}∥\frac{\partial X_{t}\left(\theta \right)}{\partial \theta }-\frac{\partial {\tilde{X}}_{t}\left(\theta \right)}{\partial \theta }∥\right)}^{2}. \end{array}$$

Using Lemma 2.1 of Straumann and Mikosch [34], together with Lemma A1, **(A2)**, **(A4)**, **(A7)**, **(A8)**, **(A10)**, and Lemma 1 of Kim and Lee [22], the right-hand side of the last inequality converges to 0 a.s. as t→∞. Hence, the first part of the lemma is verified. Similarly, we have

$$\begin{array}{cc} & \frac{1}{{\left(1+\alpha \right)}^{2}}\underset{\theta \in \Theta }{\sup}∥\frac{\partial l_{\alpha ,t}\left(\theta \right)}{\partial \theta }\frac{\partial l_{\alpha ,t}\left(\theta \right)}{\partial \theta^{T}}-\frac{\partial {\tilde{l}}_{\alpha ,t}\left(\theta \right)}{\partial \theta }\frac{\partial {\tilde{l}}_{\alpha ,t}\left(\theta \right)}{\partial \theta^{T}}∥\\ \leq & \underset{\theta \in \Theta }{\sup}∥\left(h_{\alpha }{\left(\eta_{t}\right)}^{2}-h_{\alpha }{\left({\tilde{\eta }}_{t}\right)}^{2}\right)\frac{\partial X_{t}\left(\theta \right)}{\partial \theta }\frac{\partial X_{t}\left(\theta \right)}{\partial \theta^{T}}∥+\underset{\theta \in \Theta }{\sup}∥h_{\alpha }{\left({\tilde{\eta }}_{t}\right)}^{2}\frac{\partial X_{t}\left(\theta \right)}{\partial \theta }\left(\frac{\partial X_{t}\left(\theta \right)}{\partial \theta^{T}}-\frac{\partial {\tilde{X}}_{t}\left(\theta \right)}{\partial \theta^{T}}\right)∥\\ & +\underset{\theta \in \Theta }{\sup}∥h_{\alpha }{\left({\tilde{\eta }}_{t}\right)}^{2}\left(\frac{\partial X_{t}\left(\theta \right)}{\partial \theta }-\frac{\partial {\tilde{X}}_{t}\left(\theta \right)}{\partial \theta }\right)\left(\frac{\partial {\tilde{X}}_{t}\left(\theta \right)}{\partial \theta^{T}}-\frac{\partial X_{t}\left(\theta \right)}{\partial \theta^{T}}\right)∥\\ & +\underset{\theta \in \Theta }{\sup}∥h_{\alpha }{\left({\tilde{\eta }}_{t}\right)}^{2}\left(\frac{\partial X_{t}\left(\theta \right)}{\partial \theta }-\frac{\partial {\tilde{X}}_{t}\left(\theta \right)}{\partial \theta }\right)\frac{\partial X_{t}\left(\theta \right)}{\partial \theta^{T}}∥\\ \leq & \underset{\theta \in \Theta }{\sup}\left|h_{\alpha }\left(\eta_{t}\right)-h_{\alpha }\left({\tilde{\eta }}_{t}\right)\right|\left(\underset{\theta \in \Theta }{\sup}|h_{\alpha }\left(\eta_{t}\right)|+\underset{\theta \in \Theta }{\sup}\left|h_{\alpha }\left({\tilde{\eta }}_{t}\right)\right|\right){\left(\underset{\theta \in \Theta }{\sup}∥\frac{\partial X_{t}\left(\theta \right)}{\partial \theta }∥\right)}^{2}\\ & +2\underset{\theta \in \Theta }{\sup}\left|h_{\alpha }{\left({\tilde{\eta }}_{t}\right)}^{2}\right|\underset{\theta \in \Theta }{\sup}∥\frac{\partial X_{t}\left(\theta \right)}{\partial \theta }∥\underset{\theta \in \Theta }{\sup}∥\frac{\partial X_{t}\left(\theta \right)}{\partial \theta }-\frac{\partial {\tilde{X}}_{t}\left(\theta \right)}{\partial \theta }∥\\ & +\underset{\theta \in \Theta }{\sup}\left|h_{\alpha }{\left({\tilde{\eta }}_{t}\right)}^{2}\right|{\left(\underset{\theta \in \Theta }{\sup}∥\frac{\partial X_{t}\left(\theta \right)}{\partial \theta }-\frac{\partial {\tilde{X}}_{t}\left(\theta \right)}{\partial \theta }∥\right)}^{2}, \end{array}$$

and the right-hand side of the last inequality also converges to 0 a.s. from Lemma 2.1 of Straumann and Mikosch [34]. Therefore, the lemma is asserted. □

**Lemma** **A3.**
*Suppose that conditions* **(A0)** *–* **(A11)** *hold. Then, under $H_{0}$, we have as n→∞,*

$$K_{\alpha }^{-1/2}\frac{\left[ns\right]}{\sqrt{n}}\frac{\partial {\tilde{L}}_{\alpha ,[ns]}\left(\theta_{0}\right)}{\partial \theta }\overset{w}{⟶}B_{d}\left(s\right),$$

*where $B_{d}$ is a d-dimensional Brownian motion.*

**Proof.** First, we show that $K_{\alpha }$ is nonsingular. Since $Var\left[h_{\alpha }\left(\eta_{t}^{0}\right)|F_{t-1}\right]=Var[p{\left(Y_{t}|\eta_{t}^{0}\right)}^{\alpha }\left(Y_{t}-B\left(\eta_{t}^{0}\right)\right)/B^{\prime }\left(\eta_{t}^{0}\right)$$|F_{t-1}]>0$, we have $E\left(h_{\alpha }{\left(\eta_{t}^{0}\right)}^{2}|F_{t-1}\right)>{\left[E\left(h_{\alpha }\left(\eta_{t}^{0}\right)|F_{t-1}\right)\right]}^{2}=0$. Hence, it holds that for $\nu \in R^{d}/\left\{0\right\}$,

$$\begin{array}{c} \nu^{T}K_{\alpha }\nu ={\left(1+\alpha \right)}^{2}E\left[h_{\alpha }{\left(\eta_{t}^{0}\right)}^{2}{\left(\nu^{T}\frac{\partial X_{t}\left(\theta_{0}\right)}{\partial \theta }\right)}^{2}\right]={\left(1+\alpha \right)}^{2}E\left[E\left(h_{\alpha }{\left(\eta_{t}^{0}\right)}^{2}|F_{t-1}\right){\left(\nu^{T}\frac{\partial X_{t}\left(\theta_{0}\right)}{\partial \theta }\right)}^{2}\right]>0, \end{array}$$

from **(A9)**, which implies that $K_{\alpha }$ is nonsingular. Note that

$$\begin{array}{c} E\left(\frac{\partial l_{\alpha ,t}\left(\theta_{0}\right)}{\partial \theta }|F_{t-1}\right)=\left(1+\alpha \right)\frac{\partial X_{t}\left(\theta_{0}\right)}{\partial \theta }E\left(h_{\alpha }\left(\eta_{t}^{0}\right)|F_{t-1}\right)=0, \end{array}$$

and $K_{\alpha }$ is finite from Lemma 5 of Kim and Lee [22]. Since $\partial l_{\alpha ,t}\left(\theta_{0}\right)/\partial \theta$ is stationary and ergodic, it holds from the functional central limit theorem for martingales (cf. Section 18 in Billingsley [35]) that

$$\begin{array}{c} K_{\alpha }^{-1/2}\frac{\left[ns\right]}{\sqrt{n}}\frac{\partial L_{\alpha ,[ns]}\left(\theta_{0}\right)}{\partial \theta }=K_{\alpha }^{-1/2}\frac{1}{\sqrt{n}}\sum_{t=1}^{\left[ns\right]}\frac{\partial l_{\alpha ,t}\left(\theta_{0}\right)}{\partial \theta }\overset{w}{⟶}B_{d}\left(s\right). \end{array}$$

Furthermore, we can show that

$$\begin{array}{c} \underset{0\leq s\leq 1}{\sup}\frac{\left[ns\right]}{\sqrt{n}}∥\frac{\partial L_{\alpha ,[ns]}\left(\theta_{0}\right)}{\partial \theta }-\frac{\partial {\tilde{L}}_{\alpha ,[ns]}\left(\theta_{0}\right)}{\partial \theta }∥\leq \frac{1}{\sqrt{n}}\sum_{t=1}^{n}∥\frac{\partial l_{\alpha ,t}\left(\theta_{0}\right)}{\partial \theta }-\frac{\partial {\tilde{l}}_{\alpha ,t}\left(\theta_{0}\right)}{\partial \theta }∥=o\left(1\right)\;\;a.s., \end{array}$$

from Lemma 6 of Kim and Lee [22]. Hence, the lemma is verified. □

**Lemma** **A4.** *Suppose that conditions***(A0)***–***(A11)***hold. Then, under $H_{0}$, we have as n→∞,*

$$\max_{1\leq k\leq n}\frac{k}{n}∥\frac{\partial^{2}{\tilde{L}}_{\alpha ,k}\left({\bar{\theta }}_{\alpha ,n,k}\right)}{\partial \theta \partial \theta^{T}}+J_{\alpha }∥=o\left(1\right)\;\;a.s.,$$

*where $\left\{{\bar{\theta }}_{\alpha ,n,k}|1\leq k\leq n,n\geq 1\right\}$ is any double array of* Θ*-valued random vectors satisfying* $∥{\bar{\theta }}_{\alpha ,n,k}-\theta_{0}∥\leq ∥{\hat{\theta }}_{\alpha ,n}-\theta_{0}∥$*.*

**Proof.** From Lemma 5 of Kim and Lee [22], it holds that

$$\begin{array}{c} E\left(\underset{\theta \in \Theta }{\sup}∥\frac{\partial^{2}l_{\alpha ,t}\left(\theta \right)}{\partial \theta \partial \theta^{T}}-\frac{\partial^{2}l_{\alpha ,t}\left(\theta_{0}\right)}{\partial \theta \partial \theta^{T}}∥\right)<\infty . \end{array}$$

Since $\partial^{2}l_{\alpha ,t}\left(\theta \right)/\partial \theta \partial \theta^{T}$ is continuous in θ, for any ϵ>0, we can take a neighborhood $N_{\epsilon }\left(\theta_{0}\right)$, such that

(A1) $$\begin{array}{c} E\left(\underset{\theta \in N_{\epsilon }\left(\theta_{0}\right)}{\sup}∥\frac{\partial^{2}l_{\alpha ,t}\left(\theta \right)}{\partial \theta \partial \theta^{T}}-\frac{\partial^{2}l_{\alpha ,t}\left(\theta_{0}\right)}{\partial \theta \partial \theta^{T}}∥\right)<\epsilon \end{array}$$

by decreasing the neighborhood to $\theta_{0}$. Since ${\hat{\theta }}_{\alpha ,n}$ converges to $\theta_{0}$ a.s. by Proposition 1, we can write that for sufficiently large *n*,

$$\begin{array}{cc} & \max_{1\leq k\leq n}\frac{k}{n}∥\frac{\partial^{2}{\tilde{L}}_{\alpha ,k}\left({\bar{\theta }}_{\alpha ,n,k}\right)}{\partial \theta \partial \theta^{T}}+J_{\alpha }∥\\ \leq & \max_{1\leq k\leq n}\frac{k}{n}∥\frac{\partial^{2}{\tilde{L}}_{\alpha ,k}\left({\bar{\theta }}_{\alpha ,n,k}\right)}{\partial \theta \partial \theta^{T}}-\frac{\partial^{2}L_{\alpha ,k}\left({\bar{\theta }}_{\alpha ,n,k}\right)}{\partial \theta \partial \theta^{T}}∥+\max_{1\leq k\leq n}\frac{k}{n}∥\frac{\partial^{2}L_{\alpha ,k}\left({\bar{\theta }}_{\alpha ,n,k}\right)}{\partial \theta \partial \theta^{T}}-\frac{\partial^{2}L_{\alpha ,k}\left(\theta_{0}\right)}{\partial \theta \partial \theta^{T}}∥\\ & +\max_{1\leq k\leq n}\frac{k}{n}∥\frac{\partial^{2}L_{\alpha ,k}\left(\theta_{0}\right)}{\partial \theta \partial \theta^{T}}+J_{\alpha }∥\\ \leq & \frac{1}{n}\sum_{t=1}^{n}\underset{\theta \in N_{\epsilon }\left(\theta_{0}\right)}{\sup}∥\frac{\partial^{2}{\tilde{l}}_{\alpha ,t}\left(\theta \right)}{\partial \theta \partial \theta^{T}}-\frac{\partial^{2}l_{\alpha ,t}\left(\theta \right)}{\partial \theta \partial \theta^{T}}∥+\frac{1}{n}\sum_{t=1}^{n}\underset{\theta \in N_{\epsilon }\left(\theta_{0}\right)}{\sup}∥\frac{\partial^{2}l_{\alpha ,t}\left(\theta \right)}{\partial \theta \partial \theta^{T}}-\frac{\partial^{2}l_{\alpha ,t}\left(\theta_{0}\right)}{\partial \theta \partial \theta^{T}}∥\\ & +\max_{1\leq k\leq n}\frac{k}{n}∥\frac{\partial^{2}L_{\alpha ,k}\left(\theta_{0}\right)}{\partial \theta \partial \theta^{T}}+J_{\alpha }∥\\ := & I_{n}+II_{n}+III_{n}\;\;a.s. \end{array}$$

By Lemma A2, $I_{n}=o\left(1\right)$ a.s. By using (A1) and the stationarity and ergodicity of $\partial^{2}l_{\alpha ,t}\left(\theta \right)/\partial \theta \partial \theta^{T}$, we have

$$\begin{array}{c} \lim_{n\to \infty }II_{n}=E\left(\underset{\theta \in N_{\epsilon }\left(\theta_{0}\right)}{\sup}∥\frac{\partial^{2}l_{\alpha ,t}\left(\theta \right)}{\partial \theta \partial \theta^{T}}-\frac{\partial^{2}l_{\alpha ,t}\left(\theta_{0}\right)}{\partial \theta \partial \theta^{T}}∥\right)<\epsilon \;\;a.s. \end{array}$$

Finally, since $∥\partial^{2}L_{\alpha ,n}\left(\theta_{0}\right)/\partial \theta \partial \theta^{T}+J_{\alpha }∥$ converges to 0 a.s., we can show that

$$\begin{array}{c} \max_{1\leq k\leq \sqrt{n}}\frac{k}{n}∥\frac{\partial^{2}L_{\alpha ,k}\left(\theta_{0}\right)}{\partial \theta \partial \theta^{T}}+J_{\alpha }∥\leq \frac{1}{\sqrt{n}}\underset{1\leq k}{\sup}∥\frac{\partial^{2}L_{\alpha ,k}\left(\theta_{0}\right)}{\partial \theta \partial \theta^{T}}+J_{\alpha }∥=o\left(1\right)\;\;a.s., \end{array}$$

and

$$\begin{array}{c} \max_{\sqrt{n}\leq k\leq n}\frac{k}{n}∥\frac{\partial^{2}L_{\alpha ,k}\left(\theta_{0}\right)}{\partial \theta \partial \theta^{T}}+J_{\alpha }∥\leq \max_{\sqrt{n}\leq k\leq n}∥\frac{\partial^{2}L_{\alpha ,k}\left(\theta_{0}\right)}{\partial \theta \partial \theta^{T}}+J_{\alpha }∥=o\left(1\right)\;\;a.s., \end{array}$$

which assert $III_{n}=o\left(1\right)$ a.s. Therefore, the lemma is established. □

**Proof of Theorem** **1.** First, we show that

(A2) $$\begin{array}{c} \frac{\left[ns\right]}{\sqrt{n}}\frac{\partial {\tilde{L}}_{\alpha ,[ns]}\left(\theta_{0}\right)}{\partial \theta }+\frac{\left[ns\right]}{n}\frac{\partial^{2}{\tilde{L}}_{\alpha ,[ns]}\left(\theta_{\alpha ,n,s}^{*}\right)}{\partial \theta \partial \theta^{T}}J_{\alpha }^{-1}\sqrt{n}\frac{\partial {\tilde{L}}_{\alpha ,n}\left(\theta_{0}\right)}{\partial \theta }\overset{w}{⟶}K_{\alpha }^{1/2}B_{d}^{o}\left(s\right). \end{array}$$

From Lemma A3, we have

$$\begin{array}{c} \frac{\left[ns\right]}{\sqrt{n}}\frac{\partial {\tilde{L}}_{\alpha ,[ns]}\left(\theta_{0}\right)}{\partial \theta }-\frac{\left[ns\right]}{n}\sqrt{n}\frac{\partial {\tilde{L}}_{\alpha ,n}\left(\theta_{0}\right)}{\partial \theta }\overset{w}{⟶}K_{\alpha }^{1/2}B_{d}^{o}\left(s\right). \end{array}$$

Since $\sqrt{n}\partial {\tilde{L}}_{\alpha ,n}\left(\theta_{0}\right)/\partial \theta =O_{p}\left(1\right)$ by Lemma A3 with s=1, using Lemma A4, it holds that

$$\begin{array}{cc} & \underset{0\leq s\leq 1}{\sup}\frac{\left[ns\right]}{n}∥\frac{\partial^{2}{\tilde{L}}_{\alpha ,[ns]}\left(\theta_{\alpha ,n,s}^{*}\right)}{\partial \theta \partial \theta^{T}}J_{\alpha }^{-1}\sqrt{n}\frac{\partial {\tilde{L}}_{\alpha ,n}\left(\theta_{0}\right)}{\partial \theta }+\sqrt{n}\frac{\partial {\tilde{L}}_{\alpha ,n}\left(\theta_{0}\right)}{\partial \theta }∥\\ \leq & ∥J_{\alpha }^{-1}\sqrt{n}\frac{\partial {\tilde{L}}_{\alpha ,n}\left(\theta_{0}\right)}{\partial \theta }∥\max_{1\leq k\leq n}\frac{k}{n}∥\frac{\partial^{2}{\tilde{L}}_{\alpha ,k}\left(\theta_{\alpha ,n,k}^{*}\right)}{\partial \theta \partial \theta^{T}}+J_{\alpha }∥\\ = & o_{p}\left(1\right), \end{array}$$

where $\theta_{\alpha ,n,k}^{*}$ denotes that corresponding to $\theta_{\alpha ,n,s}^{*}$ when [ns]=k. Hence, (A2) is verified. Next, from Lemma A4, we have

$$\begin{array}{c} \underset{0\leq s\leq 1}{\sup}\frac{\left[ns\right]}{n}∥\frac{\partial^{2}{\tilde{L}}_{\alpha ,[ns]}\left(\theta_{\alpha ,n,s}^{*}\right)}{\partial \theta \partial \theta^{T}}∥\leq \max_{1\leq k\leq n}\frac{k}{n}∥\frac{\partial^{2}{\tilde{L}}_{\alpha ,k}\left(\theta_{\alpha ,n,k}^{*}\right)}{\partial \theta \partial \theta^{T}}+J_{\alpha }∥+∥J_{\alpha }∥=O_{p}\left(1\right) \end{array}$$

and

$$\begin{array}{c} ∥\frac{\partial^{2}{\tilde{L}}_{\alpha ,n}\left(\theta_{\alpha ,n,1}^{*}\right)}{\partial \theta \partial \theta^{T}}+J_{\alpha }∥\leq \max_{1\leq k\leq n}\frac{k}{n}∥\frac{\partial^{2}{\tilde{L}}_{\alpha ,k}\left(\theta_{\alpha ,n,k}^{*}\right)}{\partial \theta \partial \theta^{T}}+J_{\alpha }∥=o\left(1\right)\;\;a.s. \end{array}$$

Then, since $\sqrt{n}\left({\hat{\theta }}_{\alpha ,n}-\theta_{0}\right)=O_{p}\left(1\right)$ by Proposition 1, we have

(A3) $$\begin{array}{c} \underset{0\leq s\leq 1}{\sup}\frac{\left[ns\right]}{n}∥\frac{\partial^{2}{\tilde{L}}_{\alpha ,[ns]}\left(\theta_{\alpha ,n,s}^{*}\right)}{\partial \theta \partial \theta^{T}}\left(\frac{\partial^{2}{\tilde{L}}_{\alpha ,n}\left(\theta_{\alpha ,n,1}^{*}\right)}{\partial \theta \partial \theta^{T}}+J_{\alpha }\right)\sqrt{n}\left({\hat{\theta }}_{\alpha ,n}-\theta_{0}\right)∥=o_{p}\left(1\right). \end{array}$$

Therefore, from (5), (A2), and (A3), the theorem is validated. □

**Lemma** **A5.**
*Suppose that conditions* **(A0)** *–* **(A11)** *hold. Then, under $H_{0}$, we have as n→∞,*

$$\frac{1}{n}\sum_{t=1}^{n}\frac{\partial {\tilde{l}}_{\alpha ,t}\left({\hat{\theta }}_{\alpha ,n}\right)}{\partial \theta }\frac{\partial {\tilde{l}}_{\alpha ,t}\left({\hat{\theta }}_{\alpha ,n}\right)}{\partial \theta^{T}}\overset{a.s.}{⟶}K_{\alpha }.$$

**Proof.** In a similar way to Lemma A4, from Lemma 5 of Kim and Lee [22], we can also take a neighborhood $N_{\epsilon }\left(\theta_{0}\right)$, such that

(A4) $$\begin{array}{cc} & \lim_{n\to \infty }\frac{1}{n}\sum_{t=1}^{n}\underset{\theta \in N_{\epsilon }\left(\theta_{0}\right)}{\sup}∥\frac{\partial l_{\alpha ,t}\left(\theta \right)}{\partial \theta }\frac{\partial l_{\alpha ,t}\left(\theta \right)}{\partial \theta^{T}}-\frac{\partial l_{\alpha ,t}\left(\theta_{0}\right)}{\partial \theta }\frac{\partial l_{\alpha ,t}\left(\theta_{0}\right)}{\partial \theta^{T}}∥\\ = & E\left(\underset{\theta \in N_{\epsilon }\left(\theta_{0}\right)}{\sup}∥\frac{\partial l_{\alpha ,t}\left(\theta \right)}{\partial \theta }\frac{\partial l_{\alpha ,t}\left(\theta \right)}{\partial \theta^{T}}-\frac{\partial l_{\alpha ,t}\left(\theta_{0}\right)}{\partial \theta }\frac{\partial l_{\alpha ,t}\left(\theta_{0}\right)}{\partial \theta^{T}}∥\right)<\epsilon \;\;a.s. \end{array}$$

Note that we can write

$$\begin{array}{cc} & ∥\frac{1}{n}\sum_{t=1}^{n}\frac{\partial {\tilde{l}}_{\alpha ,t}\left({\hat{\theta }}_{\alpha ,n}\right)}{\partial \theta }\frac{\partial {\tilde{l}}_{\alpha ,t}\left({\hat{\theta }}_{\alpha ,n}\right)}{\partial \theta^{T}}-E\left(\frac{\partial l_{\alpha ,t}\left(\theta_{0}\right)}{\partial \theta }\frac{\partial l_{\alpha ,t}\left(\theta_{0}\right)}{\partial \theta^{T}}\right)∥\\ \leq & ∥\frac{1}{n}\sum_{t=1}^{n}\frac{\partial {\tilde{l}}_{\alpha ,t}\left({\hat{\theta }}_{\alpha ,n}\right)}{\partial \theta }\frac{\partial {\tilde{l}}_{\alpha ,t}\left({\hat{\theta }}_{\alpha ,n}\right)}{\partial \theta^{T}}-\frac{1}{n}\sum_{t=1}^{n}\frac{\partial l_{\alpha ,t}\left({\hat{\theta }}_{\alpha ,n}\right)}{\partial \theta }\frac{\partial l_{\alpha ,t}\left({\hat{\theta }}_{\alpha ,n}\right)}{\partial \theta^{T}}∥\\ & +∥\frac{1}{n}\sum_{t=1}^{n}\frac{\partial l_{\alpha ,t}\left({\hat{\theta }}_{\alpha ,n}\right)}{\partial \theta }\frac{\partial l_{\alpha ,t}\left({\hat{\theta }}_{\alpha ,n}\right)}{\partial \theta^{T}}-\frac{1}{n}\sum_{t=1}^{n}\frac{\partial l_{\alpha ,t}\left(\theta_{0}\right)}{\partial \theta }\frac{\partial l_{\alpha ,t}\left(\theta_{0}\right)}{\partial \theta^{T}}∥\\ & +∥\frac{1}{n}\sum_{t=1}^{n}\frac{\partial l_{\alpha ,t}\left(\theta_{0}\right)}{\partial \theta }\frac{\partial l_{\alpha ,t}\left(\theta_{0}\right)}{\partial \theta^{T}}-E\left(\frac{\partial l_{\alpha ,t}\left(\theta_{0}\right)}{\partial \theta }\frac{\partial l_{\alpha ,t}\left(\theta_{0}\right)}{\partial \theta^{T}}\right)∥\\ := & I_{n}+II_{n}+III_{n}. \end{array}$$

By Lemma A2,

$$\begin{array}{c} I_{n}\leq \frac{1}{n}\sum_{t=1}^{n}\underset{\theta \in \Theta }{\sup}∥\frac{\partial {\tilde{l}}_{\alpha ,t}\left(\theta \right)}{\partial \theta }\frac{\partial {\tilde{l}}_{\alpha ,t}\left(\theta \right)}{\partial \theta^{T}}-\frac{\partial l_{\alpha ,t}\left(\theta \right)}{\partial \theta }\frac{\partial l_{\alpha ,t}\left(\theta \right)}{\partial \theta^{T}}∥=o\left(1\right)\;\;a.s. \end{array}$$

Since ${\hat{\theta }}_{\alpha ,n}$ converges to $\theta_{0}$ a.s. by Proposition 1, from (A4), we have

$$\begin{array}{c} \lim_{n\to \infty }II_{n}\leq \lim_{n\to \infty }\frac{1}{n}\sum_{t=1}^{n}\underset{\theta \in N_{\epsilon }\left(\theta_{0}\right)}{\sup}∥\frac{\partial l_{\alpha ,t}\left(\theta \right)}{\partial \theta }\frac{\partial l_{\alpha ,t}\left(\theta \right)}{\partial \theta^{T}}-\frac{\partial l_{\alpha ,t}\left(\theta_{0}\right)}{\partial \theta }\frac{\partial l_{\alpha ,t}\left(\theta_{0}\right)}{\partial \theta^{T}}∥<\epsilon \;\;a.s. \end{array}$$

Finally, by the ergodic theorem, $III_{n}=o\left(1\right)$ a.s. Therefore, the lemma is established. □

## References

[1] McKenzie E. (1985). Some simple models for discrete variate time series. J. Am. Water Resour. Assoc..

[2] Al-Osh M.A., Alzaid A.A. (1987). First order integer-valued autoregressive (INAR(1)) process. J. Time Ser. Anal..

[3] Ferland R., Latour A., Oraichi D. (2006). Integer-valued GARCH processes. J. Time Ser. Anal..

[4] Engle R.F. (1982). Autoregressive conditional heteroskedasticity with estimates of the variance of United Kingdom inflation. Econometrica.

[5] Bollerslev T. (1986). Generalized autoregressive conditional heteroskedasticity. J. Econom..

[6] Fokianos K., Rahbek A., Tjøstheim D. (2009). Poisson autoregression. J. Am. Stat. Assoc..

[7] Davis R.A., Wu R. (2009). A negative binomial model for time series of counts. Biometrika.

[8] Christou V., Fokianos K. (2014). Quasi-likelihood inference for negative binomial time series models. J. Time Ser. Anal..

[9] Zhu F. (2012). Modeling overdispersed or underdispersed count data with generalized poisson integer-valued garch models. J. Math. Anal. Appl..

[10] Zhu F. (2012). Zero-inflated Poisson and negative binomial integer-valued GARCH models. J. Stat. Plan. Infer..

[11] Lee S., Lee Y., Chen C.W.S. (2016). Parameter change test for zero-inflated generalized Poisson autoregressive models. Statistics.

[12] Davis R.A., Liu H. (2016). Theory and inference for a class of observation-driven models with application to time series of counts. Stat. Sin..

[13] Diop M.L., Kengne W. (2017). Testing parameter change in general integer-valued time series. J. Time Ser. Anal..

[14] Lee Y., Lee S. (2019). CUSUM test for general nonlinear integer-valued GARCH models: Comparison study. Ann. Inst. Stat. Math..

[15] Kang J., Lee S. (2014). Parameter change test for Poisson autoregressive models. Scand. J. Stat..

[16] Basu A., Harris I.R., Hjort N.L., Jones M.C. (1998). Robust and efficient estimation by minimizing a density power divergence. Biometrika.

[17] Ghosh A., Basu A. (2013). Robust estimation for independent non-homogeneous observations using density power divergence with applications to linear regression. Electron. J. Stat..

[18] Lee S., Song J. (2009). Minimum density power divergence estimator for GARCH models. Test.

[19] Kim B., Lee S. (2013). Robust estimation for the covariance matrix of multivariate time series based on normal mixtures. Comput. Stat. Data Anal..

[20] Kang J., Lee S. (2014). Minimum density power divergence estimator for Poisson autoregressive models. Comput. Stat. Data Anal..

[21] Kim B., Lee S. (2017). Robust estimation for zero-inflated Poisson autoregressive models based on density power divergence. J. Stat. Comput. Simul..

[22] Kim B., Lee S. (2020). Robust estimation for general integer-valued time series models. Ann. Inst. Stat. Math..

[23] Kang J., Song J. (2015). Robust parameter change test for Poisson autoregressive models. Stat. Probab. Lett..

[24] Song J., Kang J. (2019). Test for parameter change in the presence of outliers: The density power divergence based approach. arXiv.

[25] Kang J., Song J. (2019). A robust approach for testing parameter change in Poisson autoregressive models. arXiv.

[26] Batsidis A., Horváth L., Martín N., Pardo L., Zografos K. (2013). Change-point detection in multinomial data using phi-divergence test statistics. J. Multivar. Anal..

[27] Batsidis A., Martín N., Pardo L., Zografos K. (2016). *ϕ*-divergence based procedure for parametric change point problems. Methodol. Comput. Appl. Probab..

[28] Martín N., Pardo L. (2014). Comment on: Extensions of some classical methods in change point analysis. Test.

[29] Lehmann E., Casella G. (1998). Theory of Point Estimation.

[30] Warwick J. (2005). A data-based method for selecting tuning parameters in minimum distance estimators. Comput. Stat. Data Anal..

[31] Warwick J., Jones M.C. (2005). Choosing a robustness tuning parameter. J. Stat. Comput. Simul..

[32] Lee S., Ha J., Na O., Na S. (2003). The cusum test for parameter change in time series models. Scand. J. Stat..

[33] Fried R., Agueusop I., Bornkamp B., Fokianos K., Fruth J., Ickstadt K. (2015). Retrospective Bayesian outlier detection in INGARCH series. Stat. Comput..

[34] Straumann D., Mikosch T. (2006). Quasi-maximum-likelihood estimation in conditionally heteroscedastic time series: A stochastic recurrence equations approach. Ann. Stat..

[35] Billingsley P. (1999). Convergence of Probability Measures.

